# Supplementary material for: VNN1 Gene Expression and Polymorphisms Associated with Chicken Carcass Traits
Source: Animals (Basel). 2024 Jun 26;14(13):1888. doi: 10.3390/ani14131888 (PMC11240768; doi:10.3390/ani14131888)
Supplement: Supplementary file 1 [file animals-14-01888-s001.zip › Supplementary File S1.pdf]

SUPPLEMENTARY FIGURES

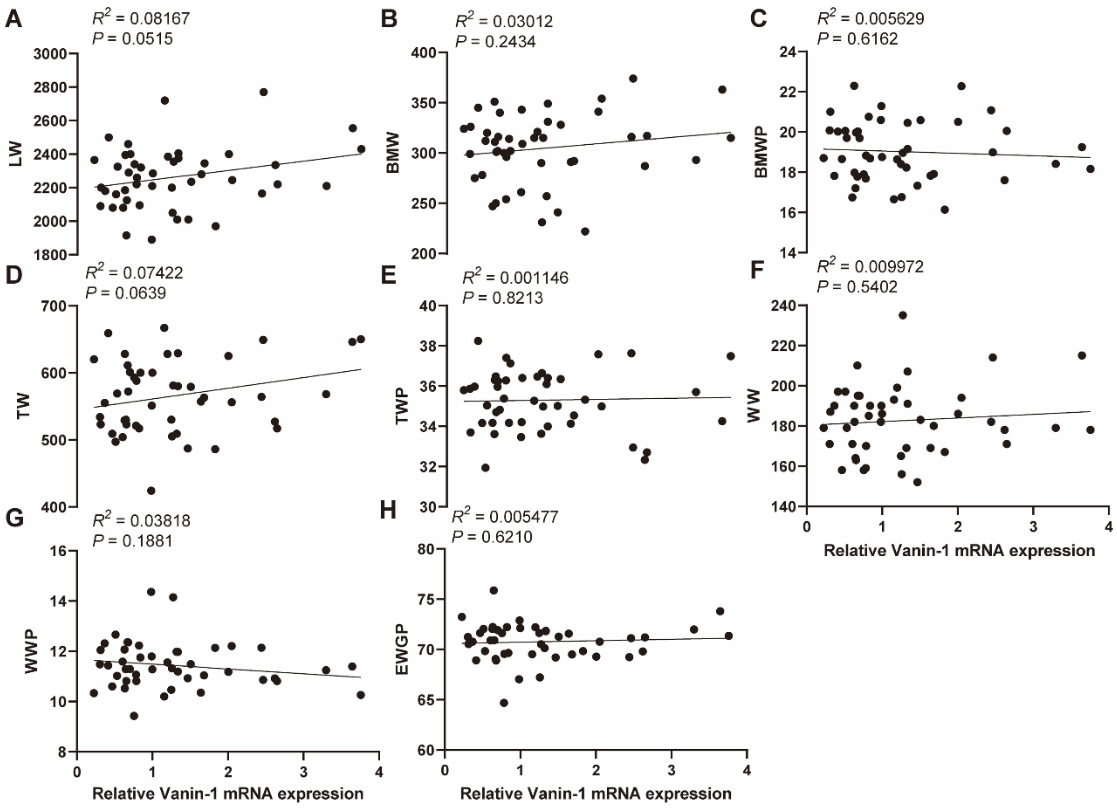

Figure S1. Association between liver *VNN1* mRNA expression and carcass traits. (A-H). Association between liver *VNN1* mRNA expression and LW, BMW, BMWP, TW, TWP, WW, WWP and EWGP in 47 female partridge chickens.

Abbreviations: LW = live weight; EWGP = eviscerated with giblet percentage; BMW = breast muscle weight; BMWP = breast muscle weight percentage; TW = thigh weight; TWP = thigh weight percentage; WW = wing weight; WWP = wing weight percentage.

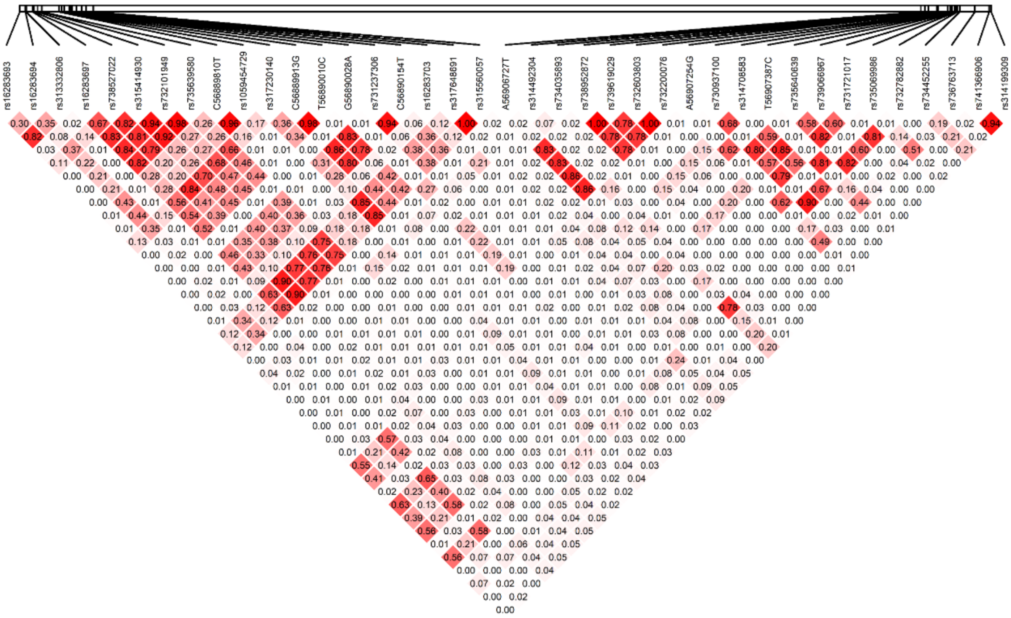

Figure S2. LD plot shows the linkage status of 39 SNPs in VNN1 gene. The color of the block indicates the LD status of SNPs; deep red means high linkages between SNPs. The number of the block show the linkages between SNPs in r2.

The figure below shows the ANOVA analysis plots for SNPs and three blocks with carcass traits. The order of the SNPs is as follows: rs16283693, rs16283694, rs313332806, rs16283697, rs738527022, rs315414930, rs732101949, rs735639580, C56889810T, rs1059454729, rs317230140, C56889913G, T56890010C, G56890028A, rs731237306, C56890154T, rs16283703, rs317648691, rs315560057, A56906727T, rs314492304, rs734035893, rs738952872, rs739619029, rs732603803, rs732200076, A56907254G, rs730937100, rs314708583, T56907387C, rs735640639, rs739066967, rs731721017, rs735069986, rs732782882, rs734452255, rs736763713, rs741366906, rs314199309, BLOCK1, BLOCK2, and BLOCK3.

The following abbreviations are used for carcass traits:

- LW(g) = live weight
- LW(%) = live weight percentage
- DW(g) = dressed weight
- DW(%) = dressed weight percentage
- EW(g) = eviscerated weight
- EW(g) = eviscerated weight percentage
- EWG(g) = eviscerated with giblet
- EWG(%) = eviscerated with giblet percentage
- BMW(g) = breast muscle weight
- BMW(%) = breast muscle weight percentage
- TW(g) = thigh weight
- TW(%) = thigh weight percentage
- WW(g) = wing weight
- WW(g) = wing weight percentage
- AFW(g) = abdominal fat weight
- AFW(%) = abdominal fat weight percentage

rs16283693

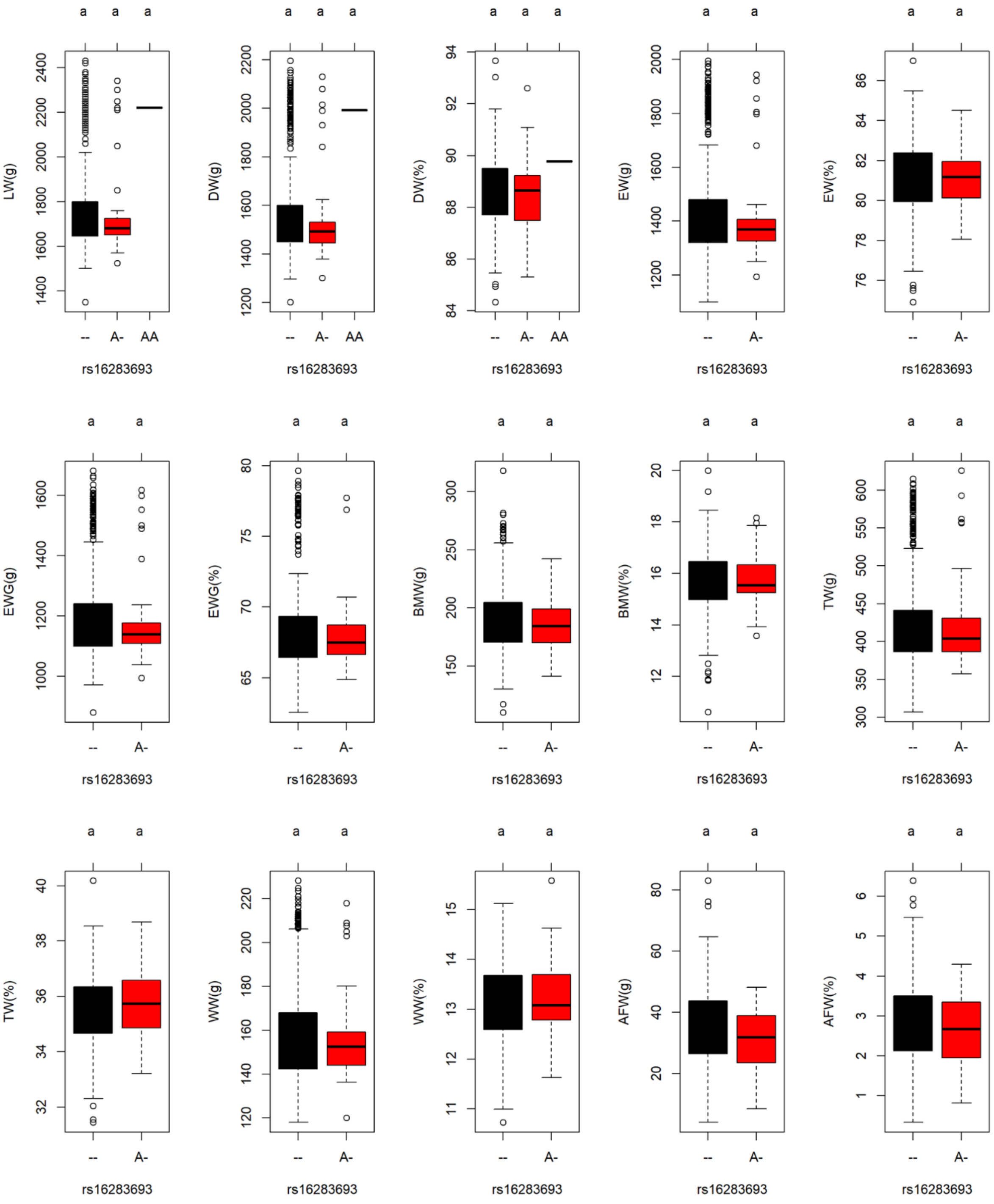
LW(g)
DW(g)
DW(%)
EW(g)
EW(%)
EWG(g)
EWG(%)
BMW(g)
BMW(%)
TW(g)
TW(%)
WW(g)
WW(%)
AFW(g)
AFW(%)
--
A-
AA
a
rs16283693

rs16283694

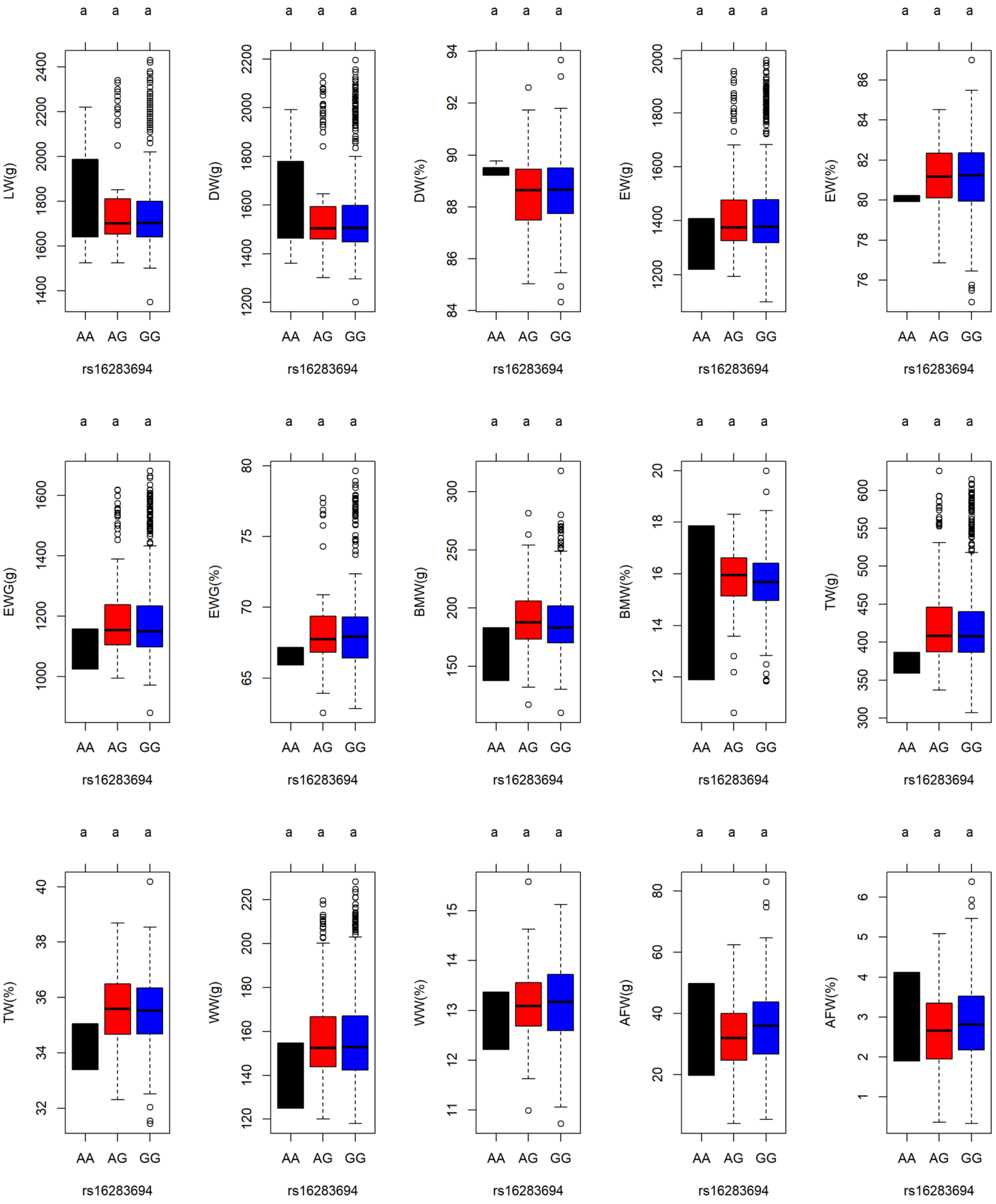
LW(g)
DW(g)
DW(%)
EW(g)
EW(%)
EWG(g)
EWG(%)
BMW(g)
BMW(%)
TW(g)
TW(%)
WW(g)
WW(%)
AFW(g)
AFW(%)
a a a
AA AG GG
rs16283694

rs313332806

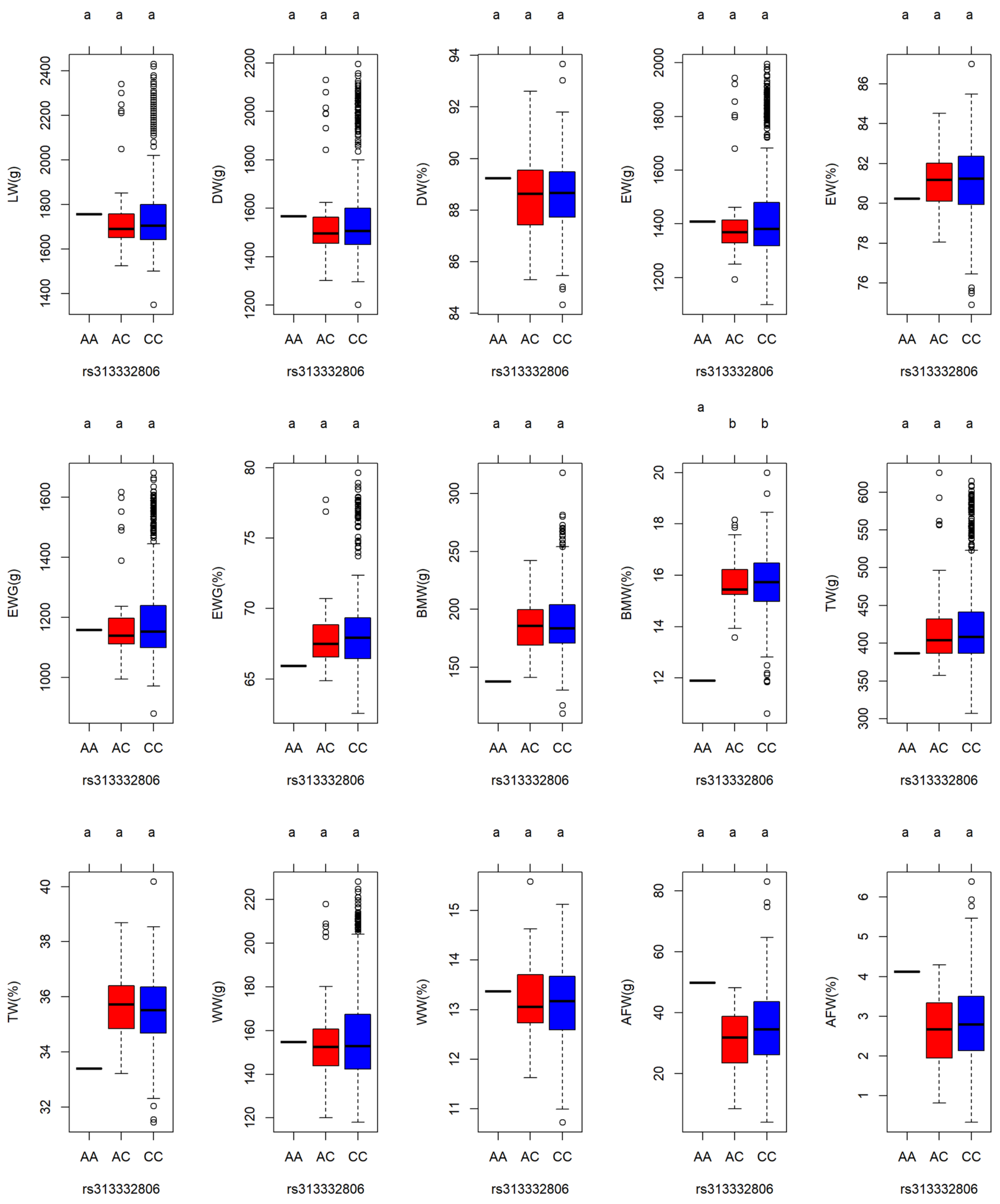

rs16283697

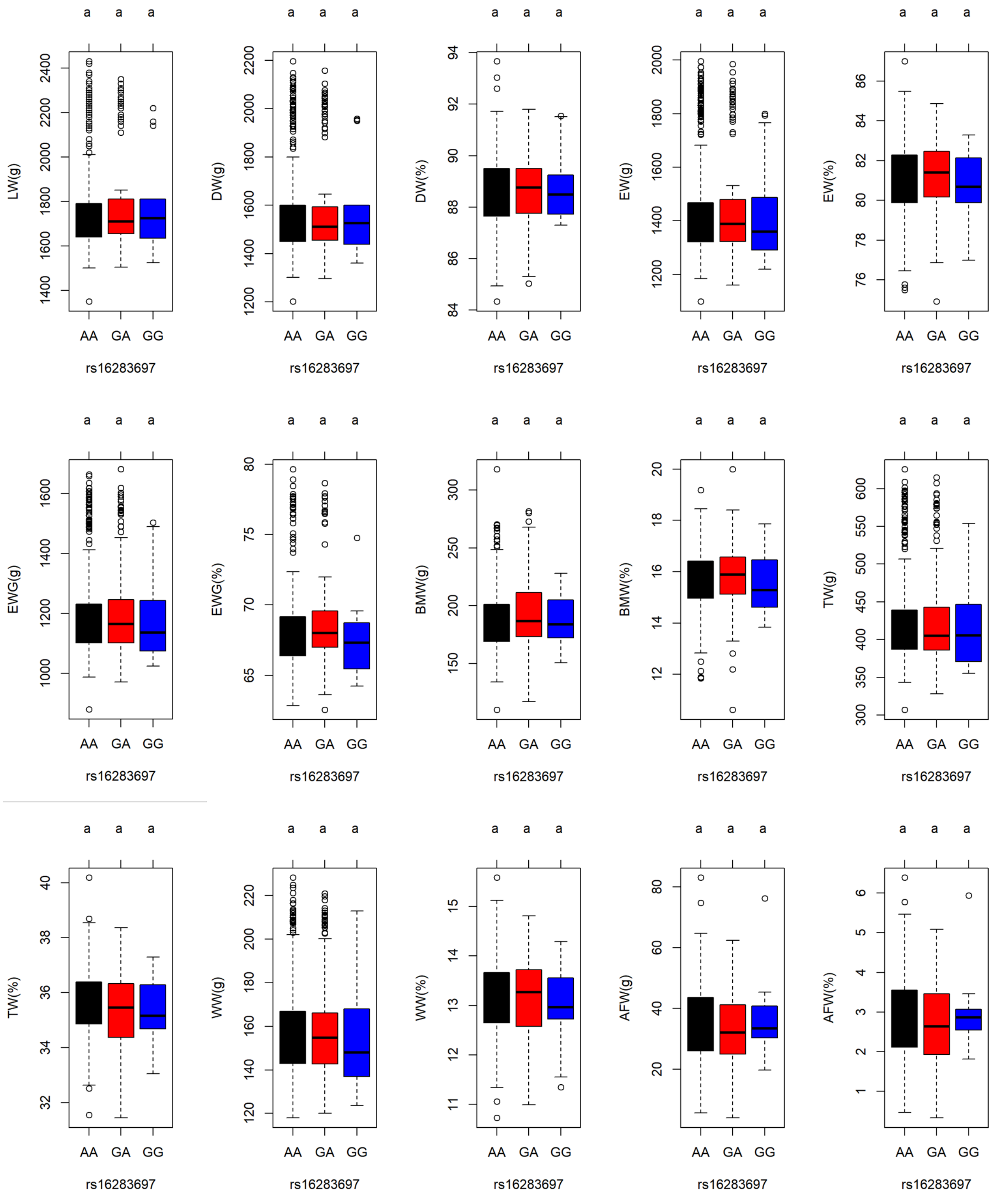

LW(g)
DW(g)
DW(%)
EW(g)
EW(%)
EWG(g)
EWG(%)
BMW(g)
BMW(%)
TW(g)
TW(%)
WW(g)
WW(%)
AFW(g)
AFW(%)
a a a
AA GA GG
rs16283697

rs738527022

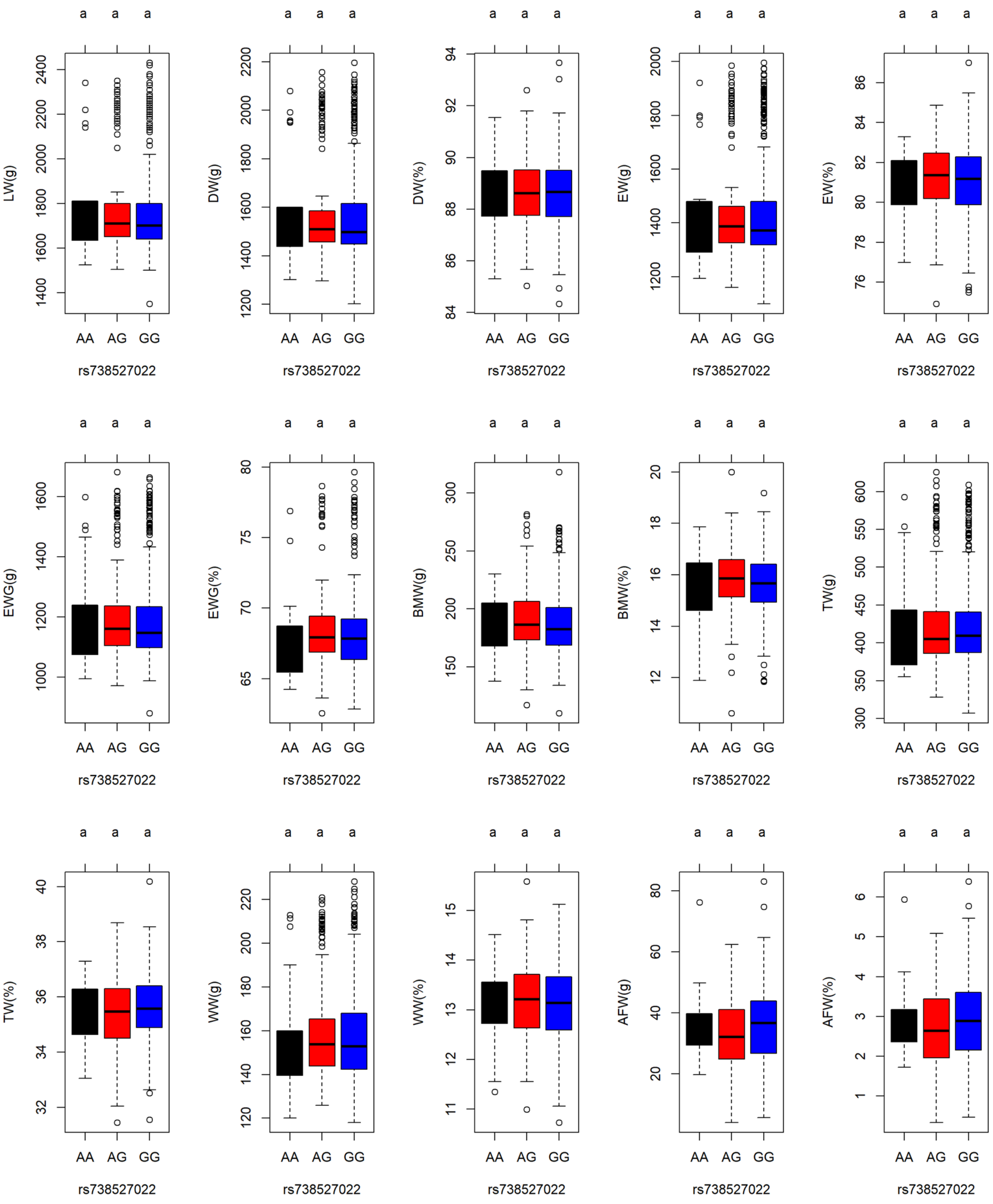

rs315414930

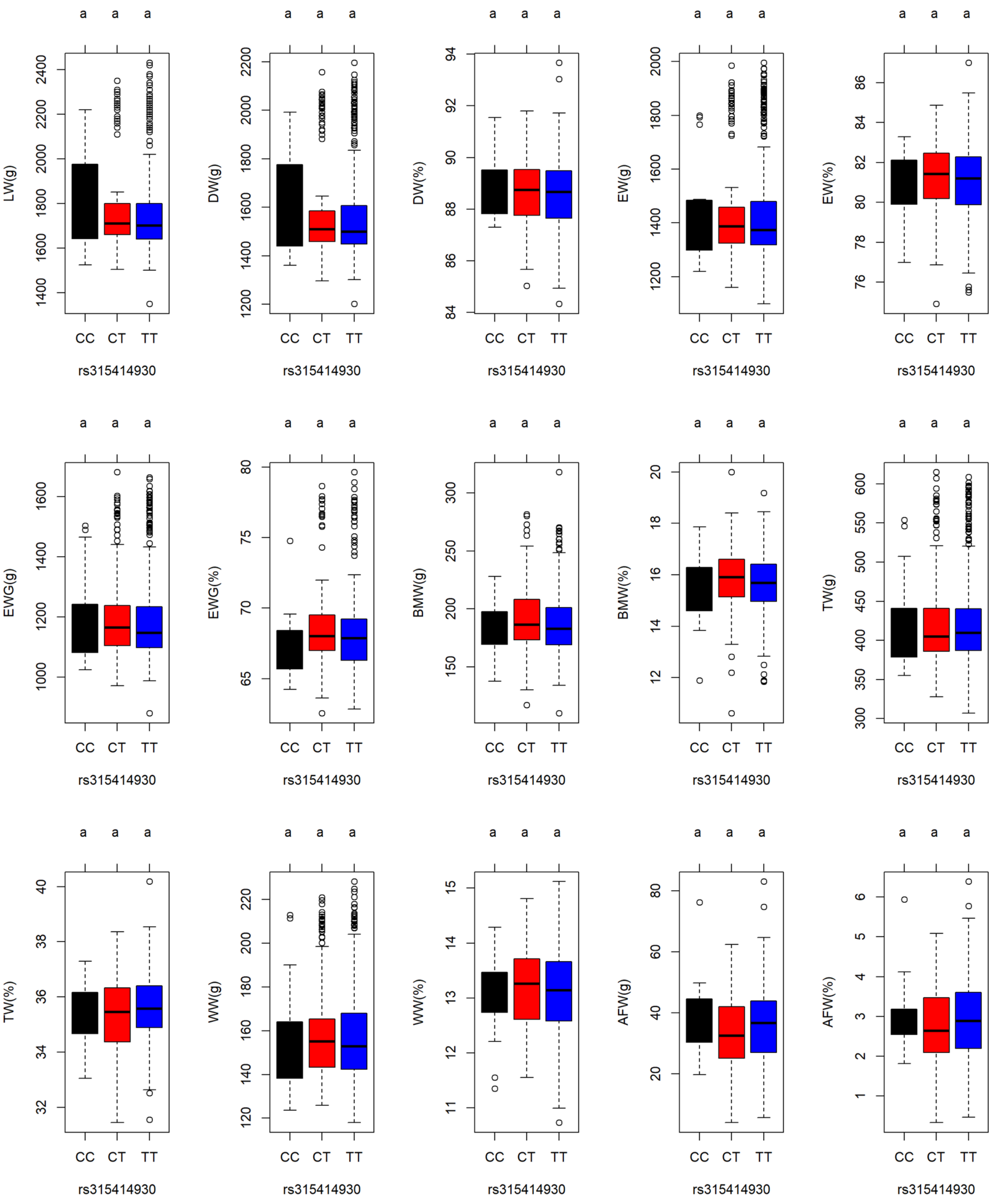

rs732101949

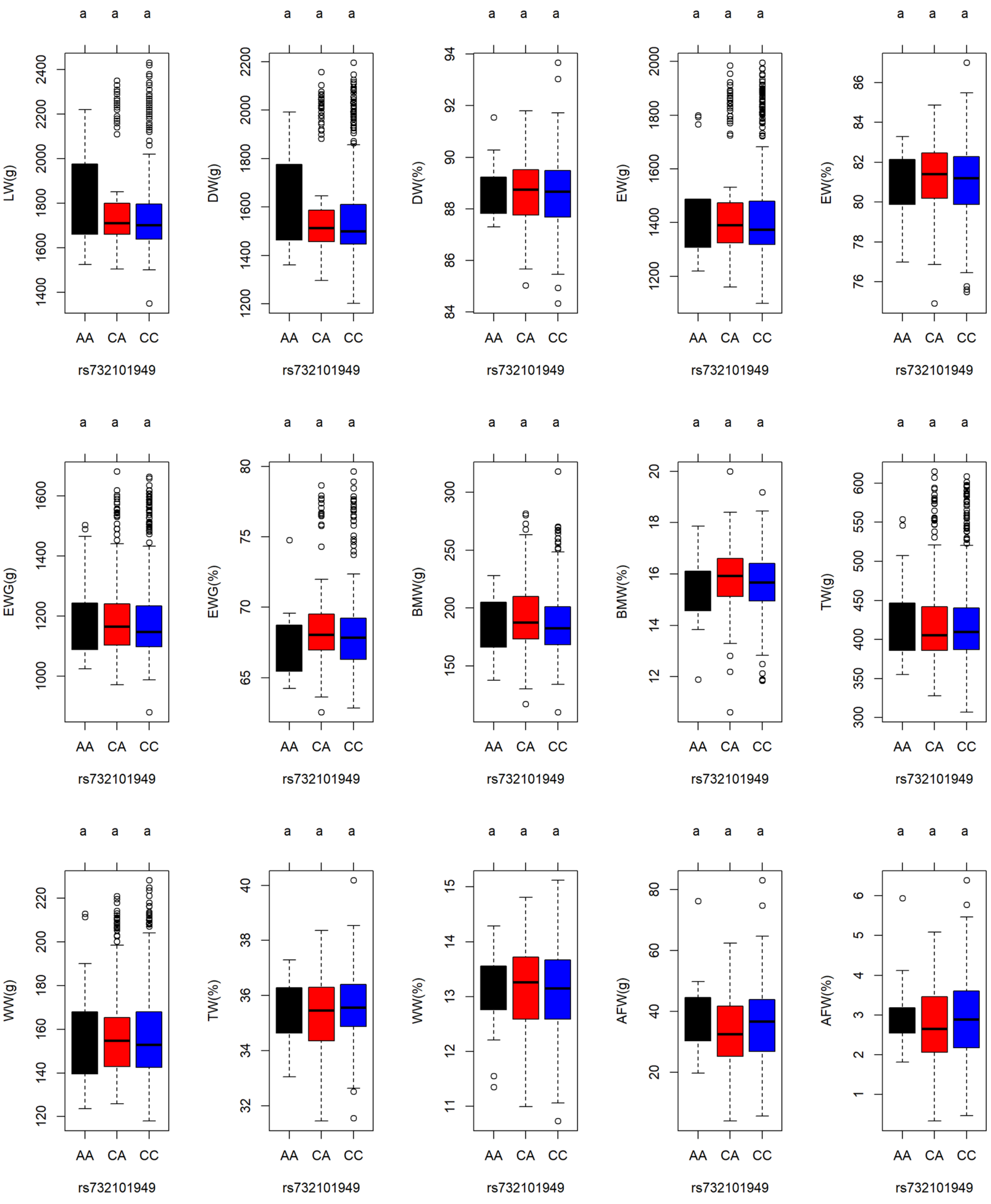

rs735639580

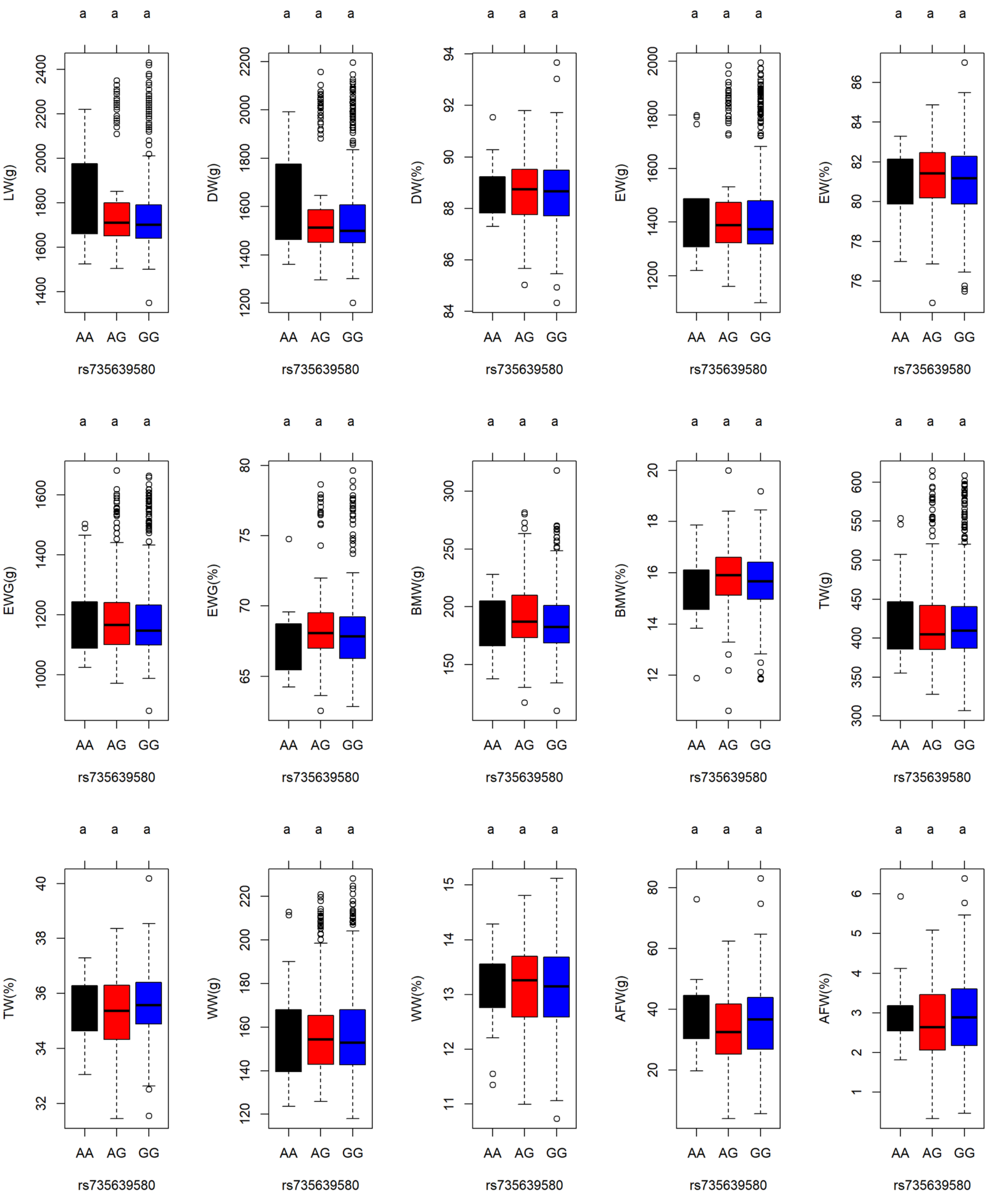

C56889810T

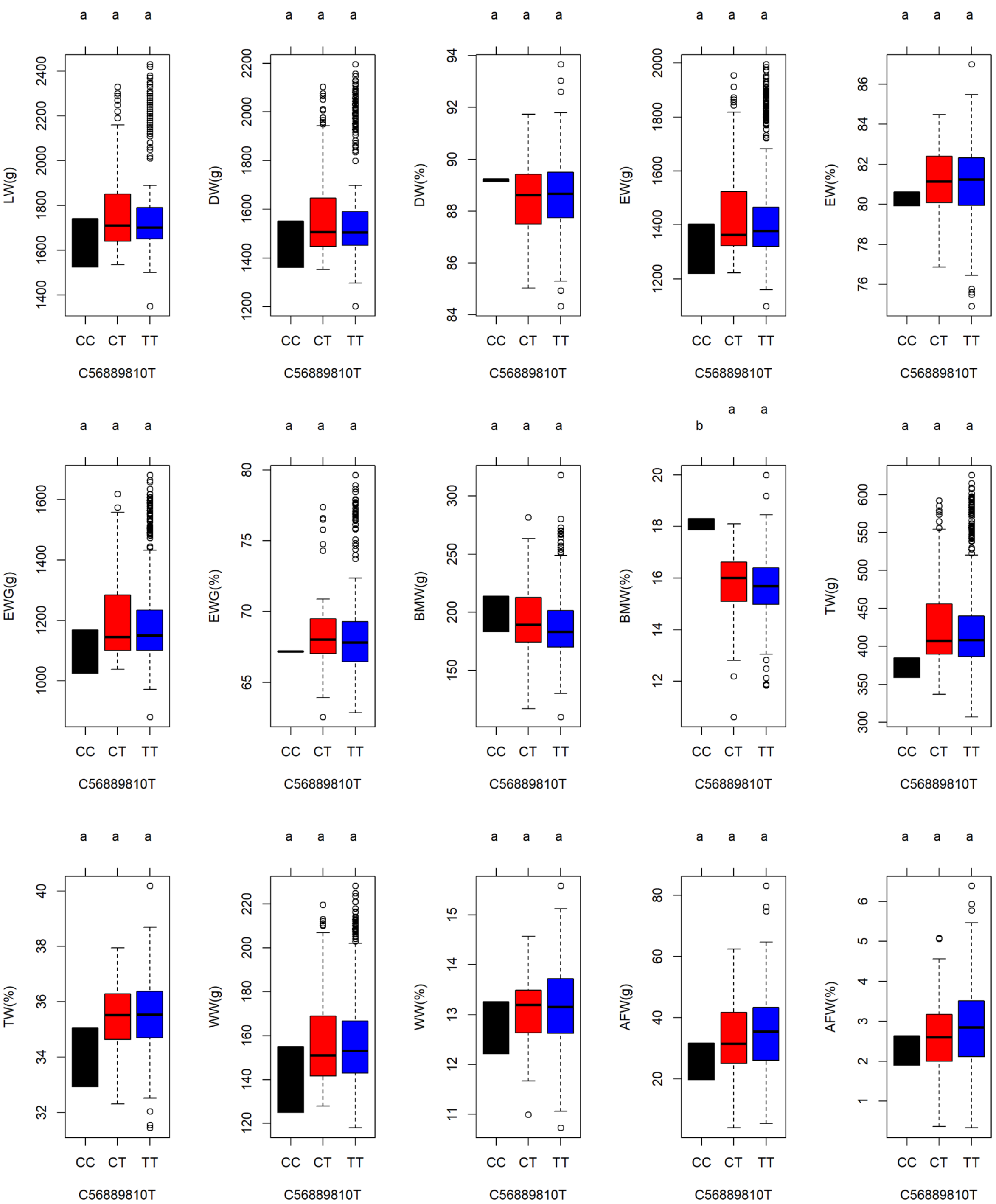

a a a
LW(g)
CC CT TT
C56889810T
a a a
DW(g)
CC CT TT
C56889810T
a a a
DW(%)
CC CT TT
C56889810T
a a a
EW(g)
CC CT TT
C56889810T
a a a
EW(%)
CC CT TT
C56889810T
a a a
EWG(g)
CC CT TT
C56889810T
a a a
EWG(%)
CC CT TT
C56889810T
a a a
BMW(g)
CC CT TT
C56889810T
b a a
BMW(%)
CC CT TT
C56889810T
a a a
TW(g)
CC CT TT
C56889810T
a a a
TW(%)
CC CT TT
C56889810T
a a a
WW(g)
CC CT TT
C56889810T
a a a
WW(%)
CC CT TT
C56889810T
a a a
AFW(g)
CC CT TT
C56889810T
a a a
AFW(%)
CC CT TT
C56889810T

rs1059454729

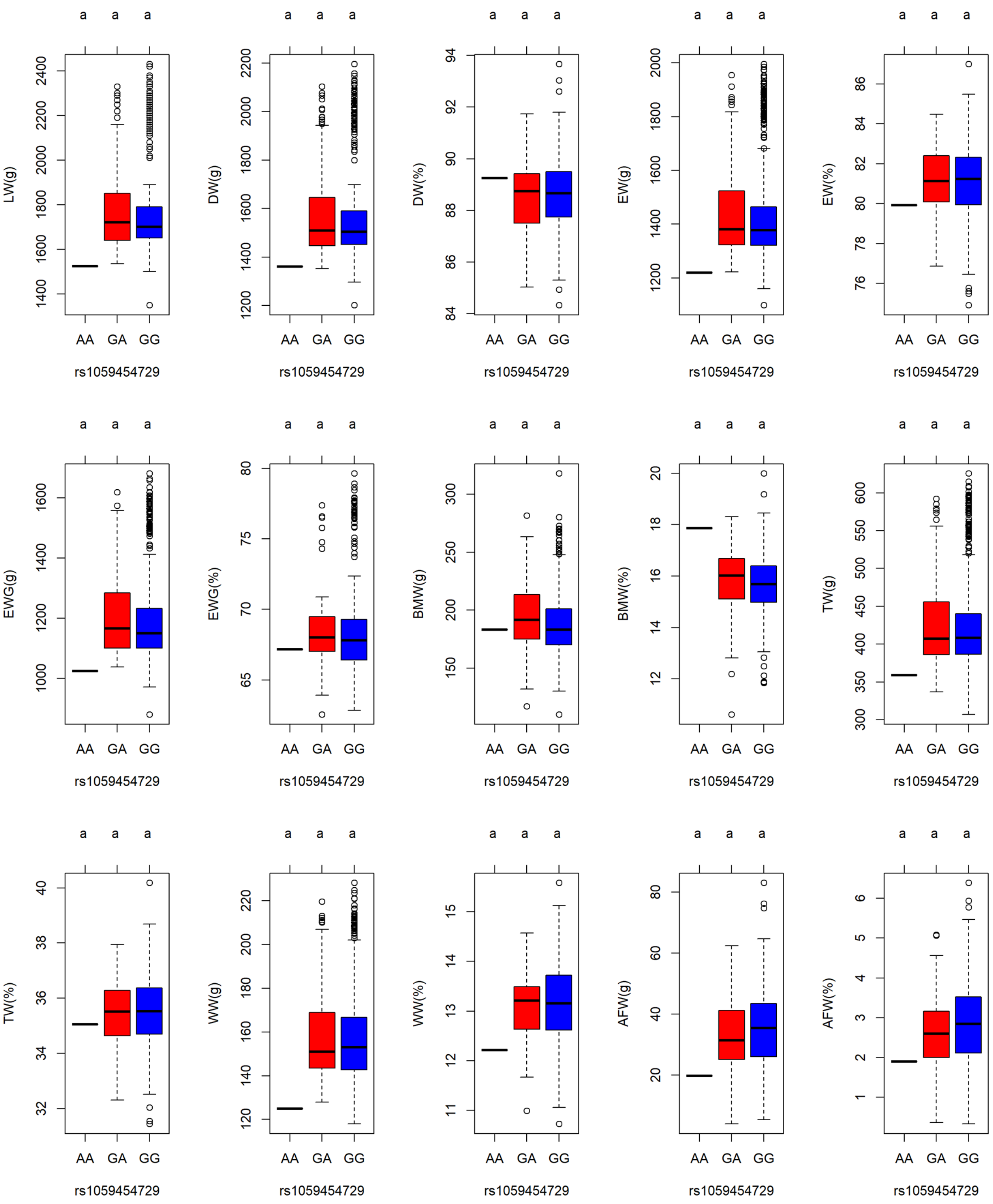

rs317230140

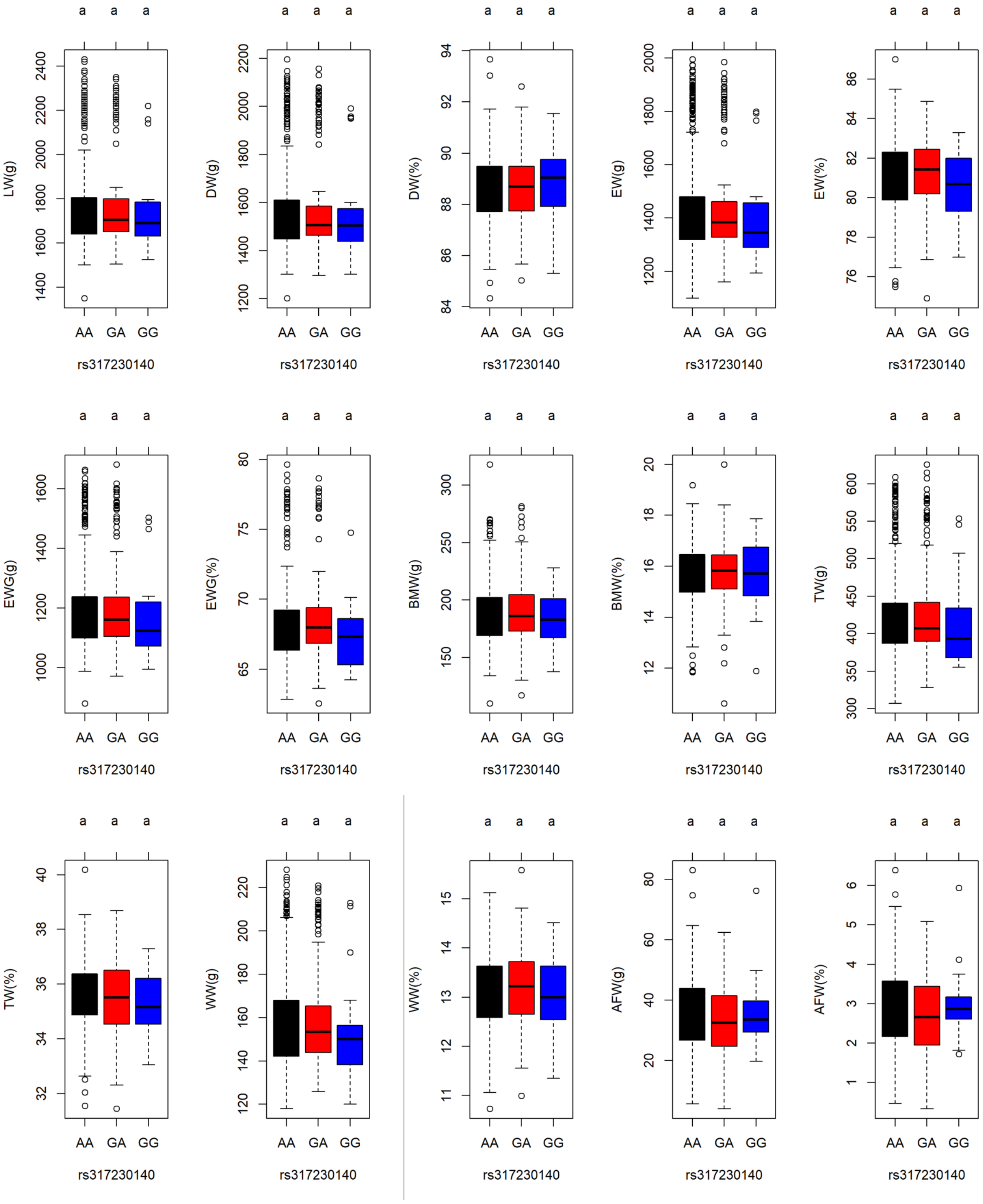

C56889913G

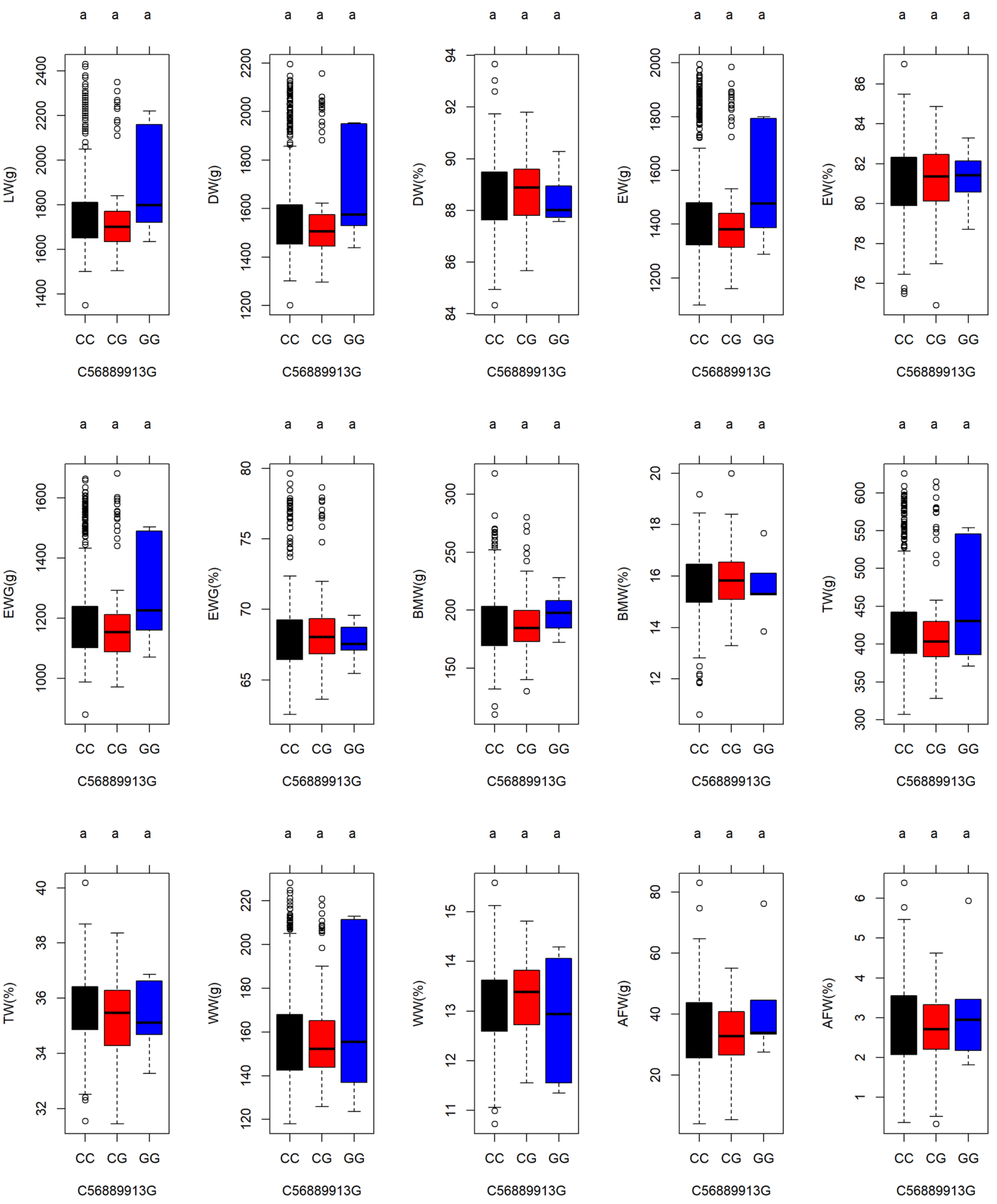

LW(g)
DW(g)
DW(%)
EW(g)
EW(%)
EWG(g)
EWG(%)
BMW(g)
BMW(%)
TW(g)
TW(%)
WW(g)
WW(%)
AFW(g)
AFW(%)
a a a
CC CG GG
C56889913G

T56890010C

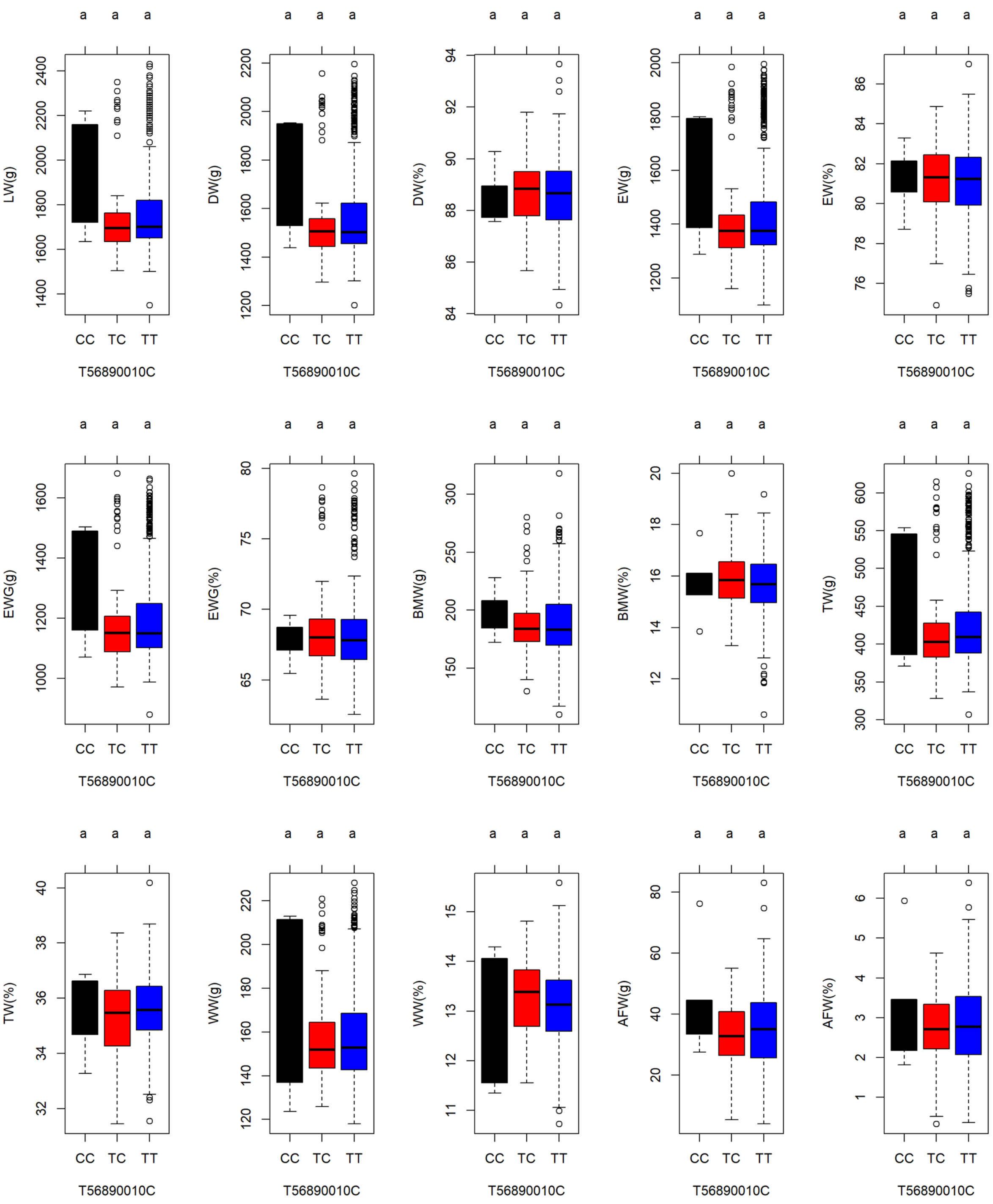

LW(g)
DW(g)
DW(%)
EW(g)
EW(%)
EWG(g)
EWG(%)
BMW(g)
BMW(%)
TW(g)
TW(%)
WW(g)
WW(%)
AFW(g)
AFW(%)
a a a
CC TC TT
T56890010C

G56890028A

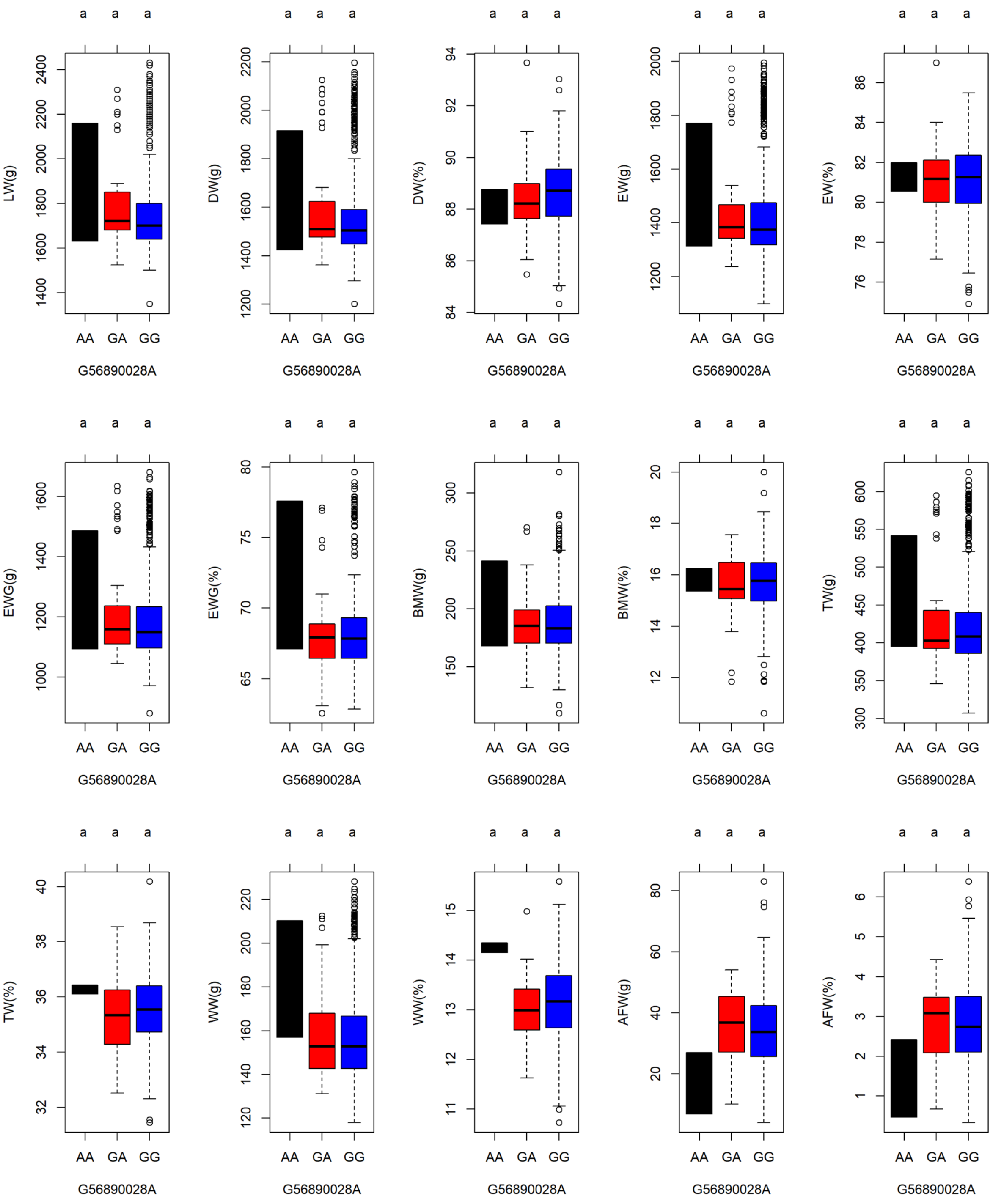
a a a
LW(g)
DW(g)
DW(%)
EW(g)
EW(%)
EWG(g)
EWG(%)
BMW(g)
BMW(%)
TW(g)
TW(%)
WW(g)
WW(%)
AFW(g)
AFW(%)
AA GA GG
G56890028A

rs731237306

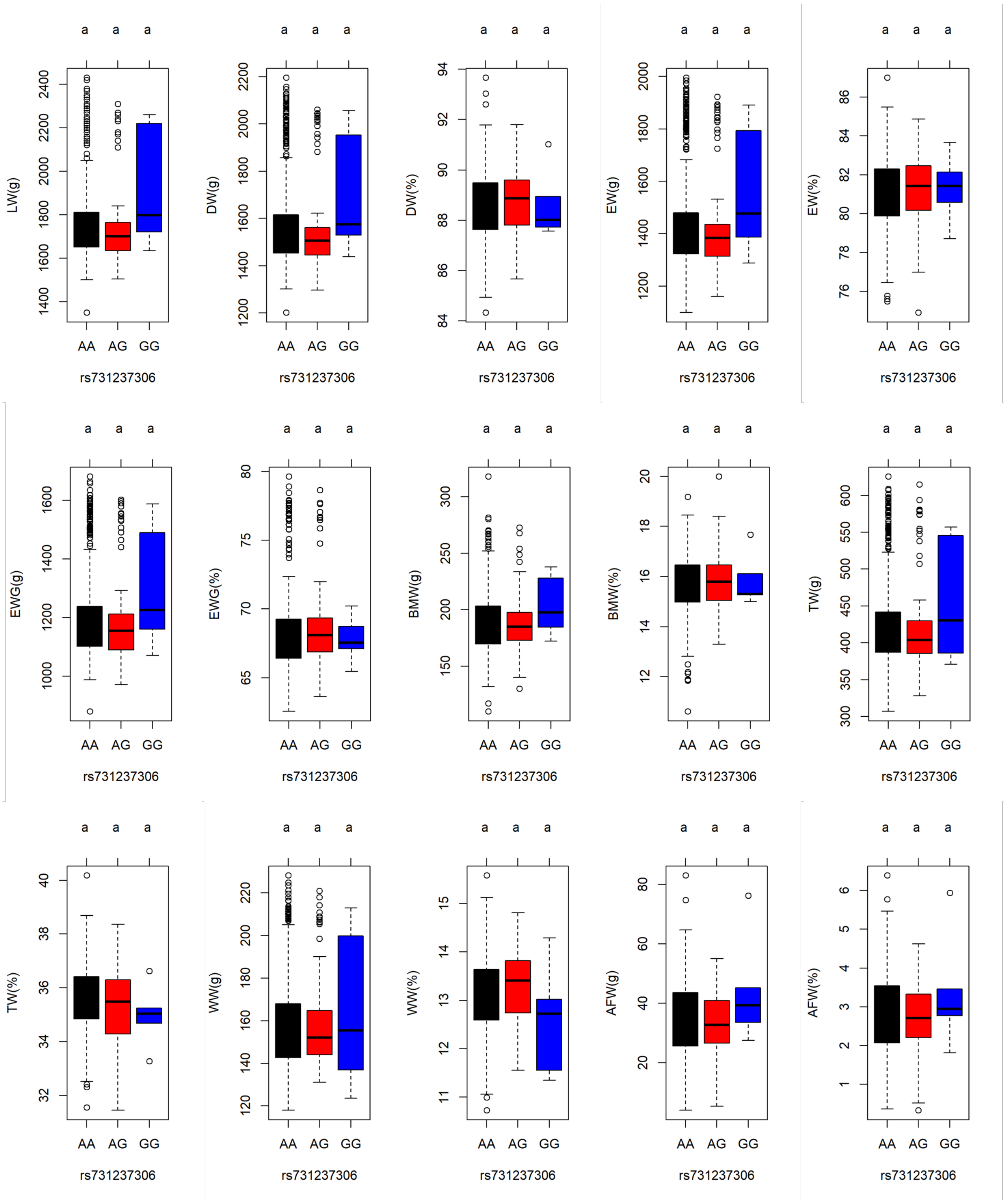

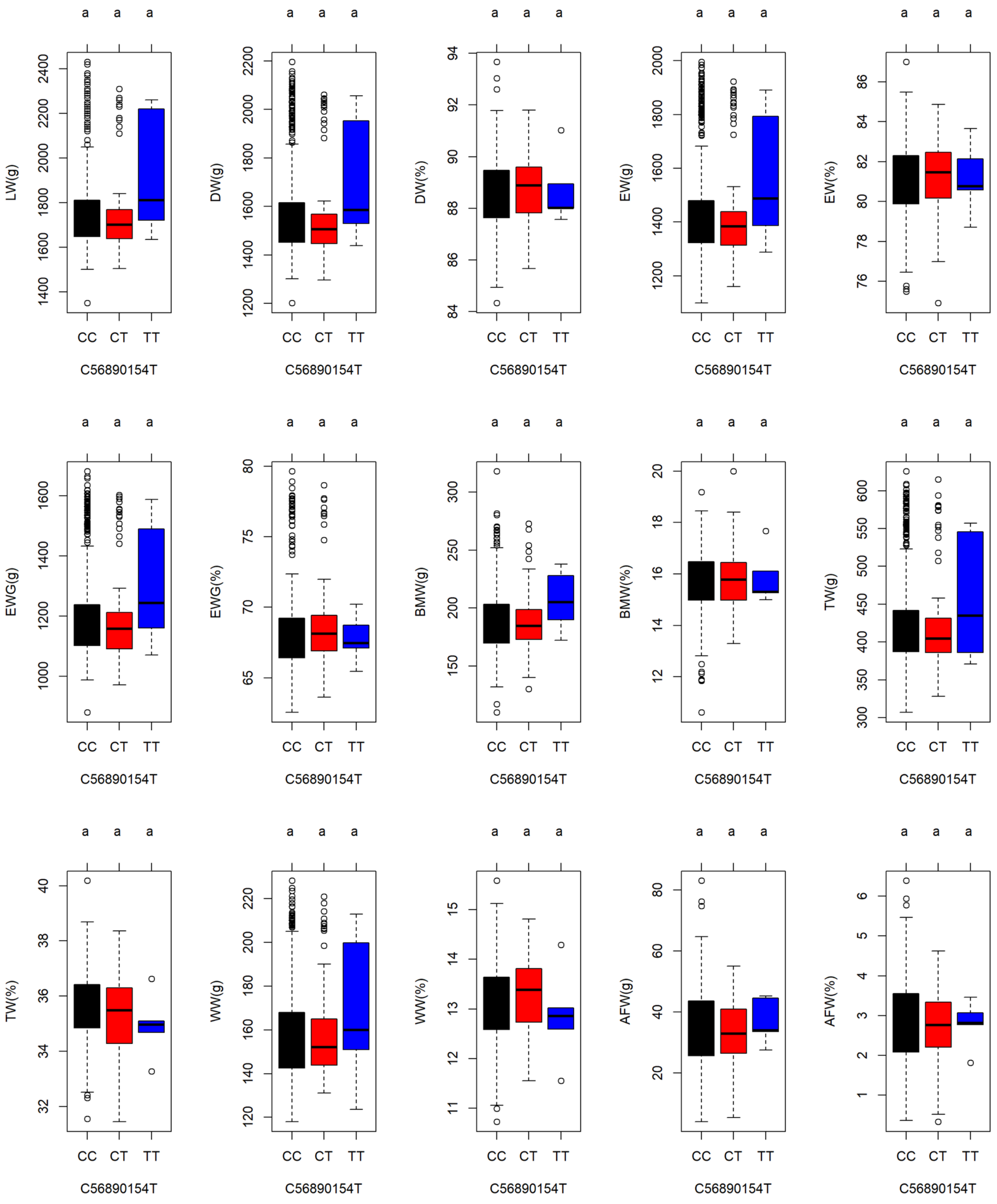
a a a
LW(g)
DW(g)
DW(%)
EW(g)
EW(%)
EWG(g)
EWG(%)
BMW(g)
BMW(%)
TW(g)
TW(%)
WW(g)
WW(%)
AFW(g)
AFW(%)
CC CT TT
C56890154T

C56890154T

rs16283703

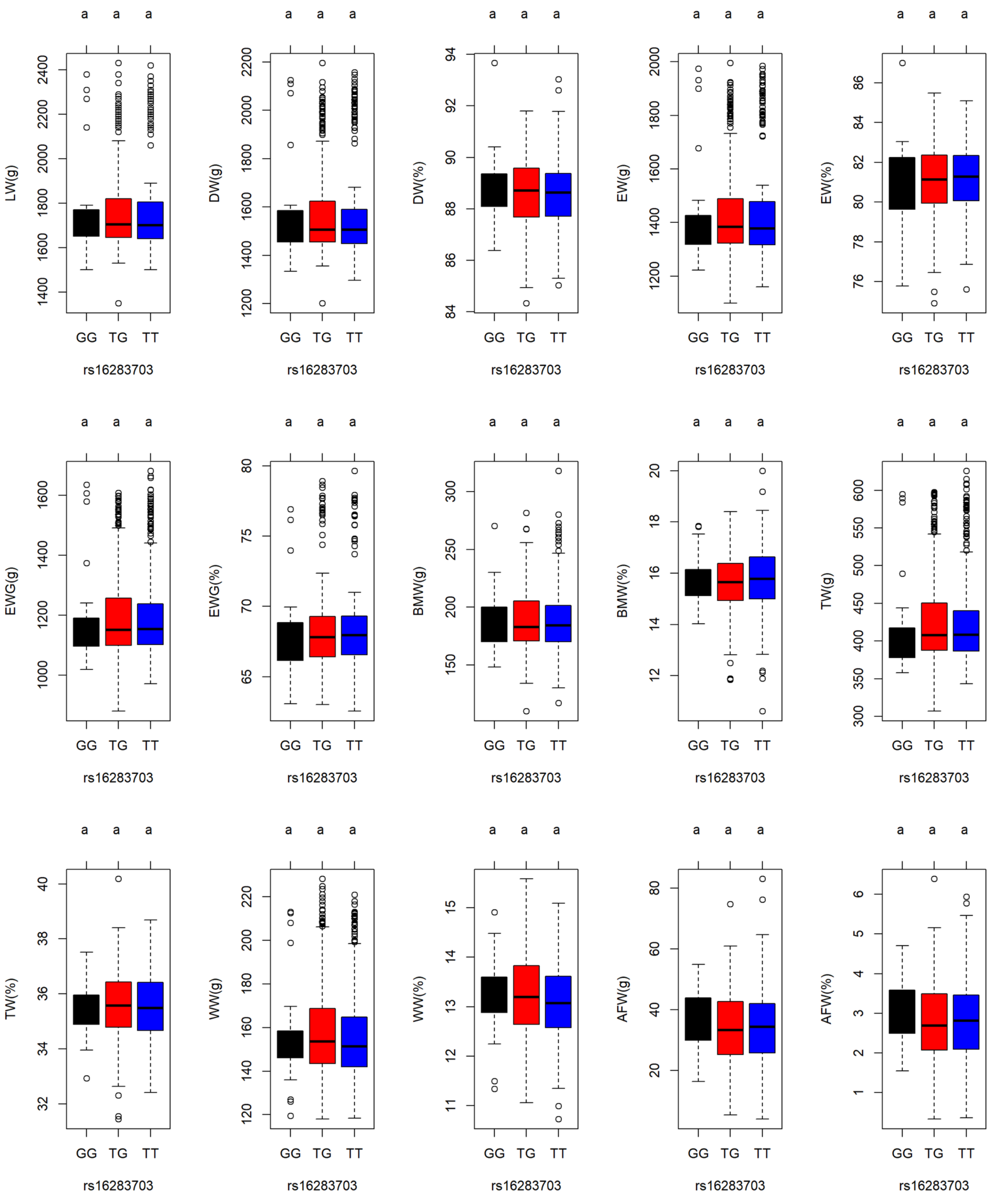
LW(g)
DW(g)
DW(%)
EW(g)
EW(%)
EWG(g)
EWG(%)
BMW(g)
BMW(%)
TW(g)
TW(%)
WW(g)
WW(%)
AFW(g)
AFW(%)
a a a
GG TG TT
rs16283703

rs317648691

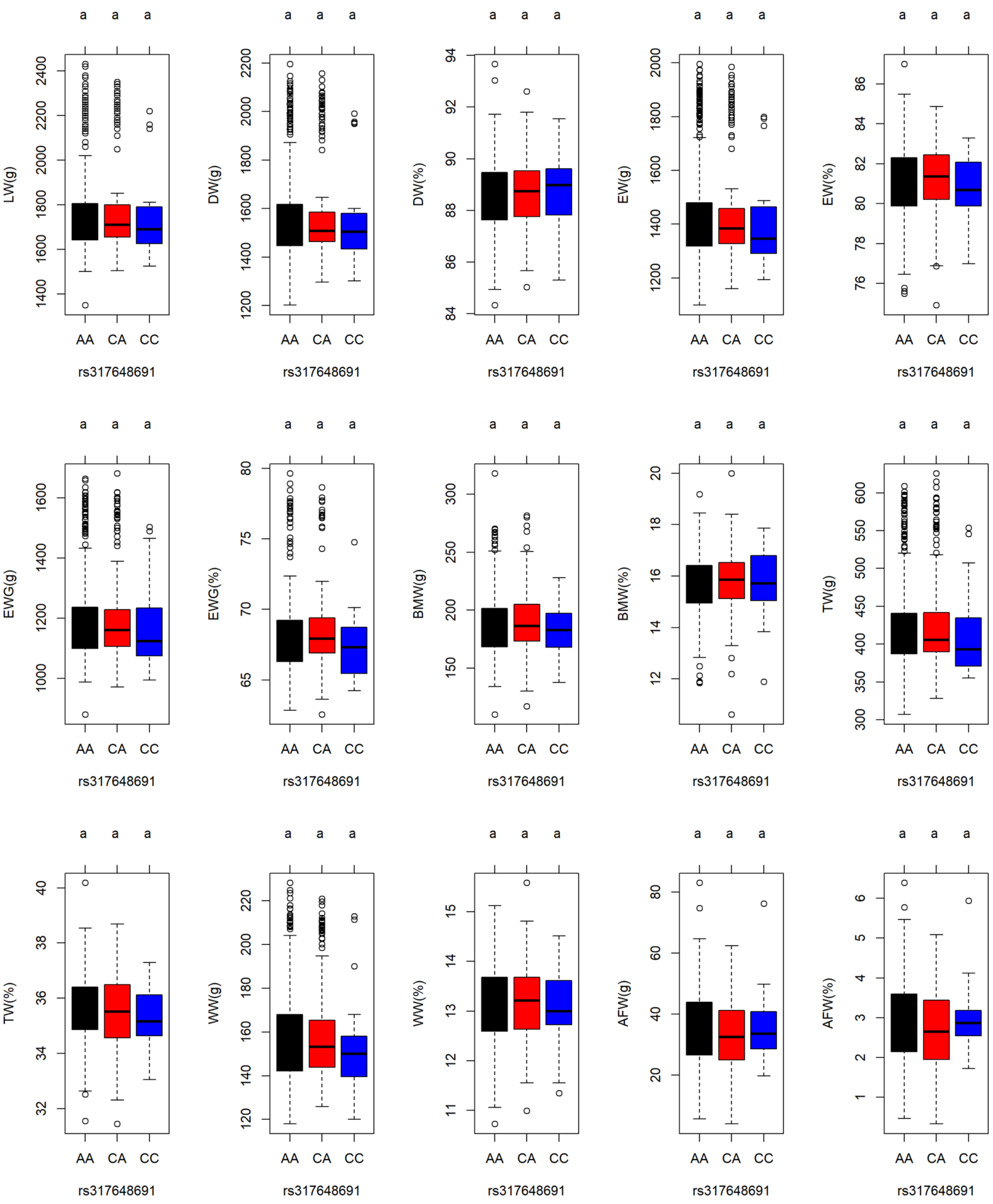

rs315560057

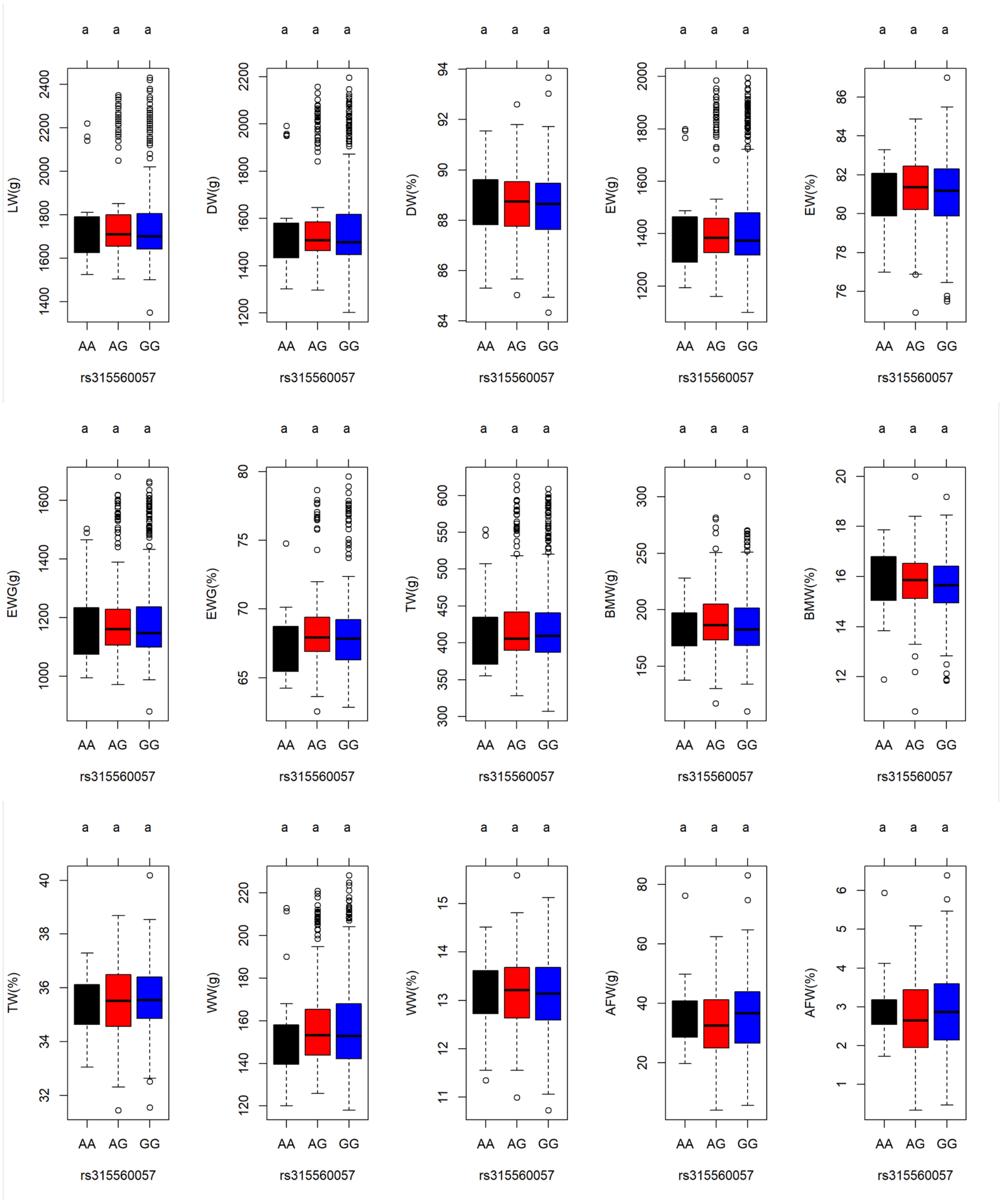

A56906727T

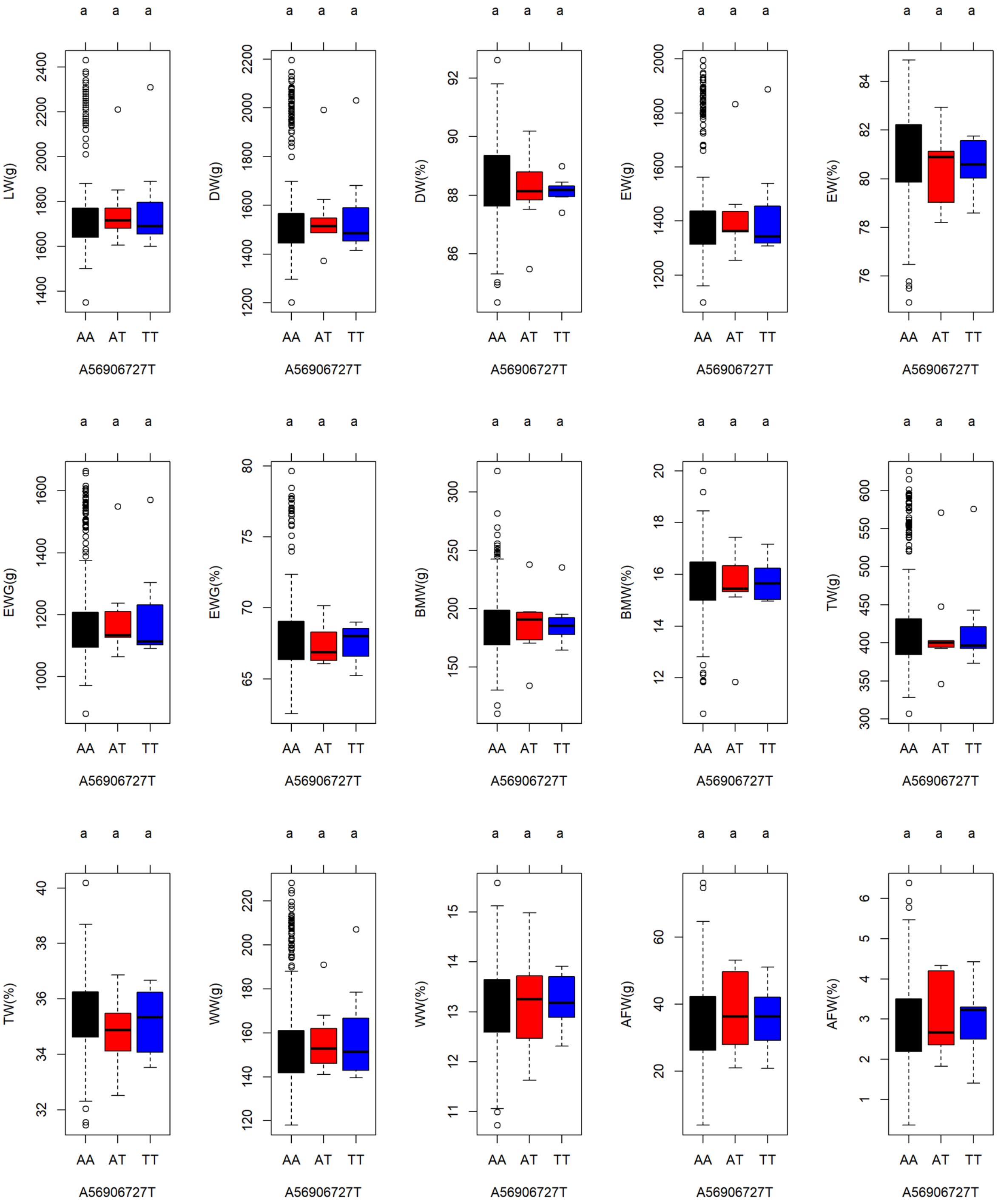

LW(g)
DW(g)
DW(%)
EW(g)
EW(%)
EWG(g)
EWG(%)
BMW(g)
BMW(%)
TW(g)
TW(%)
WW(g)
WW(%)
AFW(g)
AFW(%)
AA AT TT
A56906727T
a a a

rs314492304

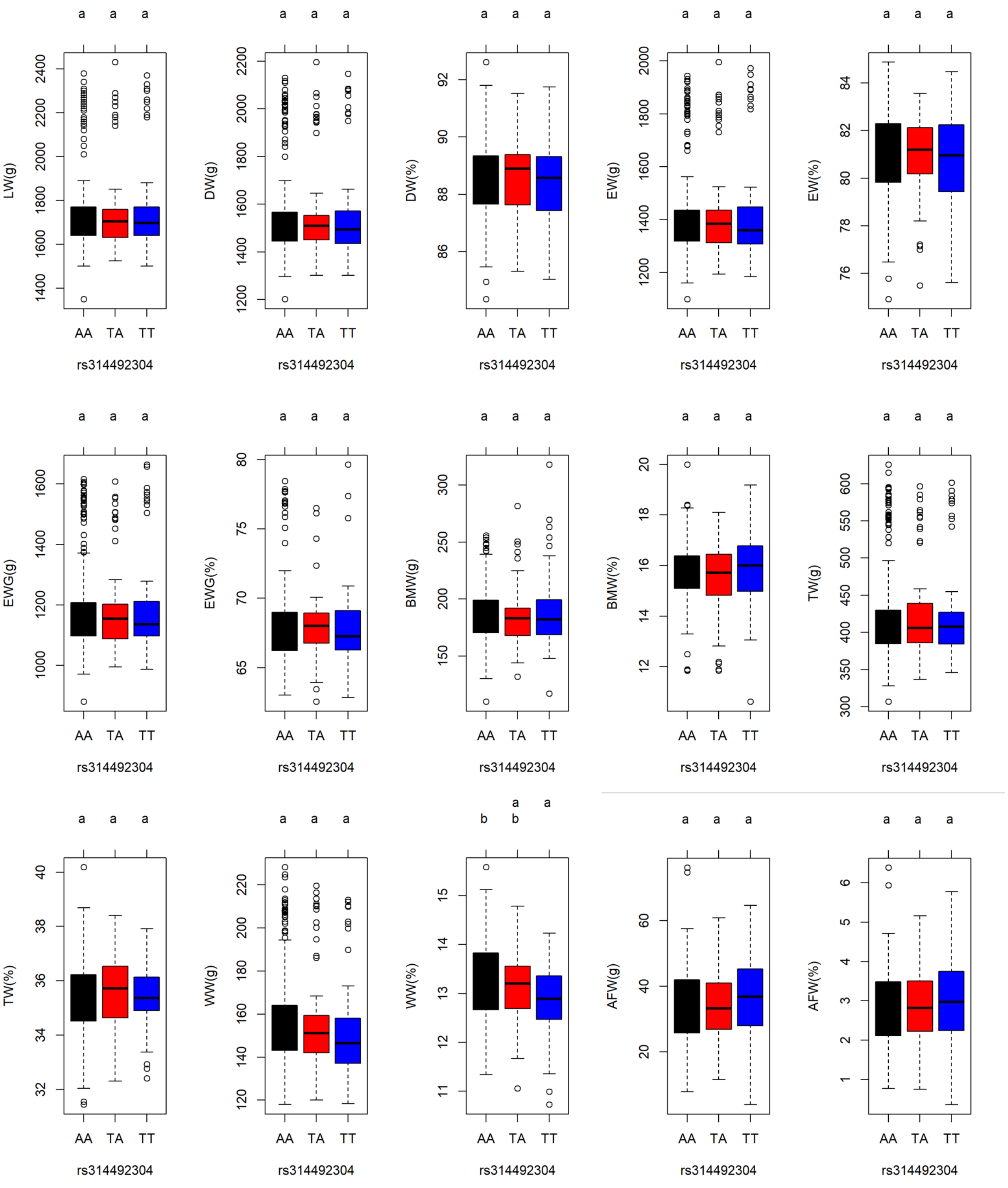

rs734035893

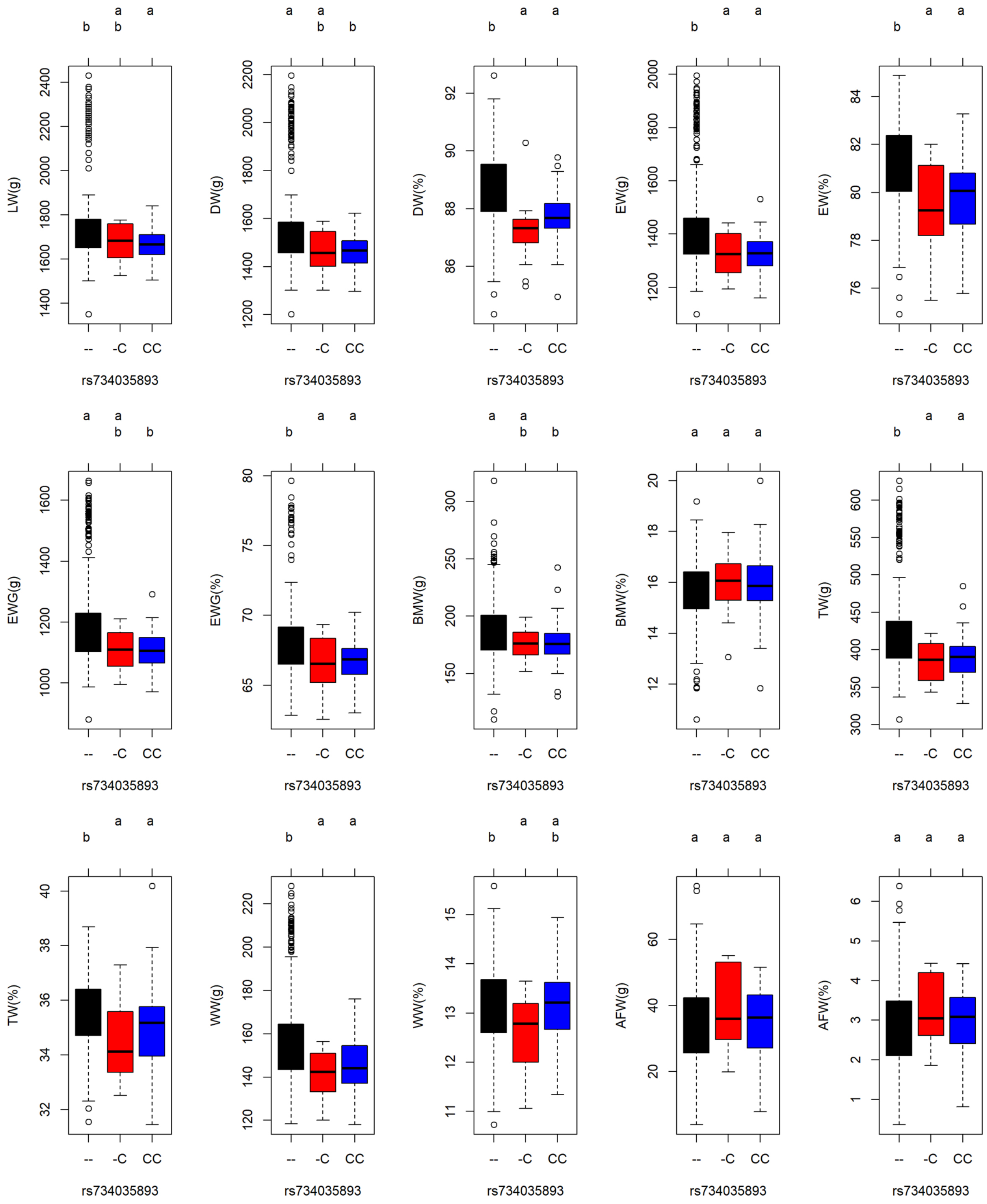

rs738952872

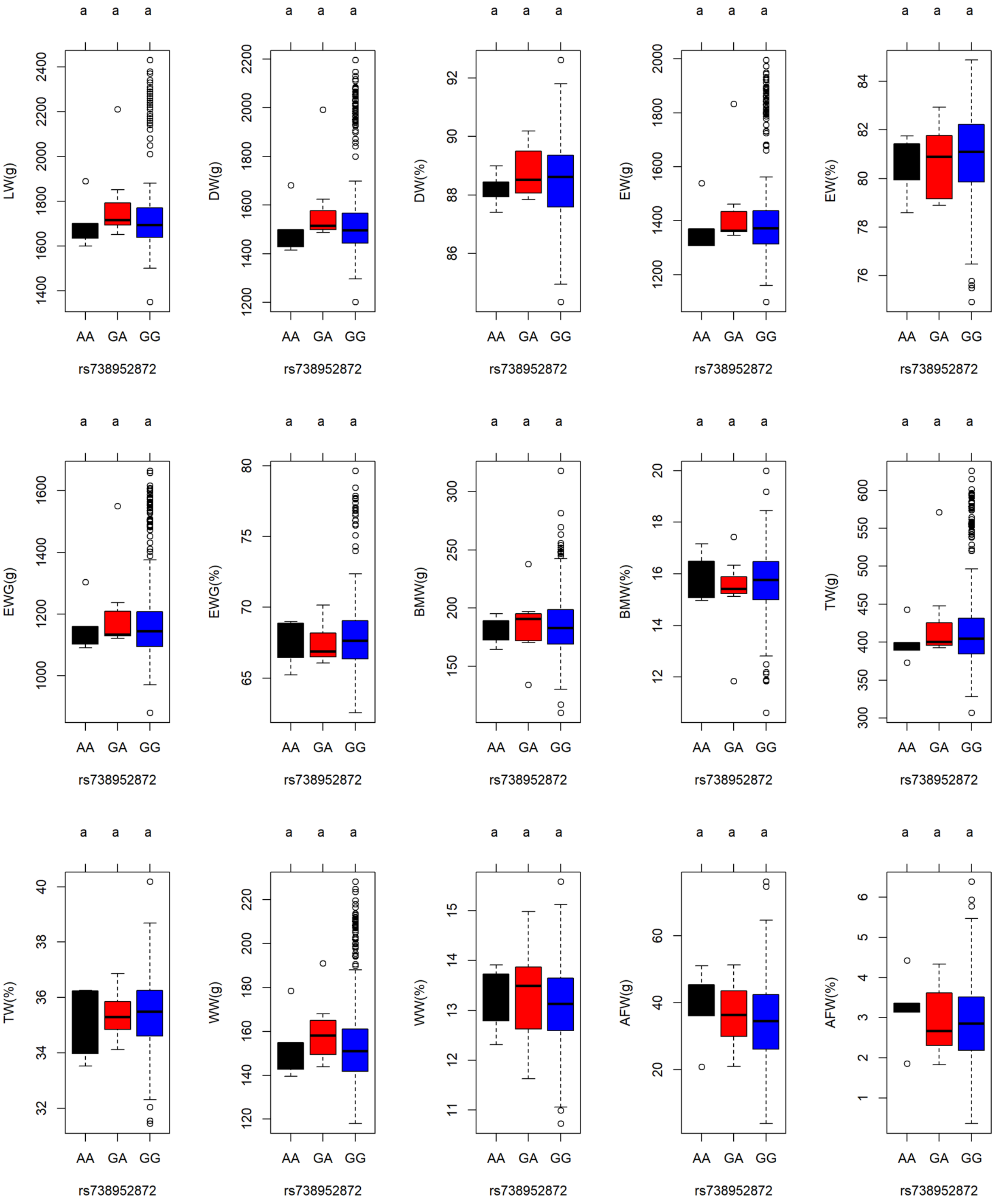

rs739619029

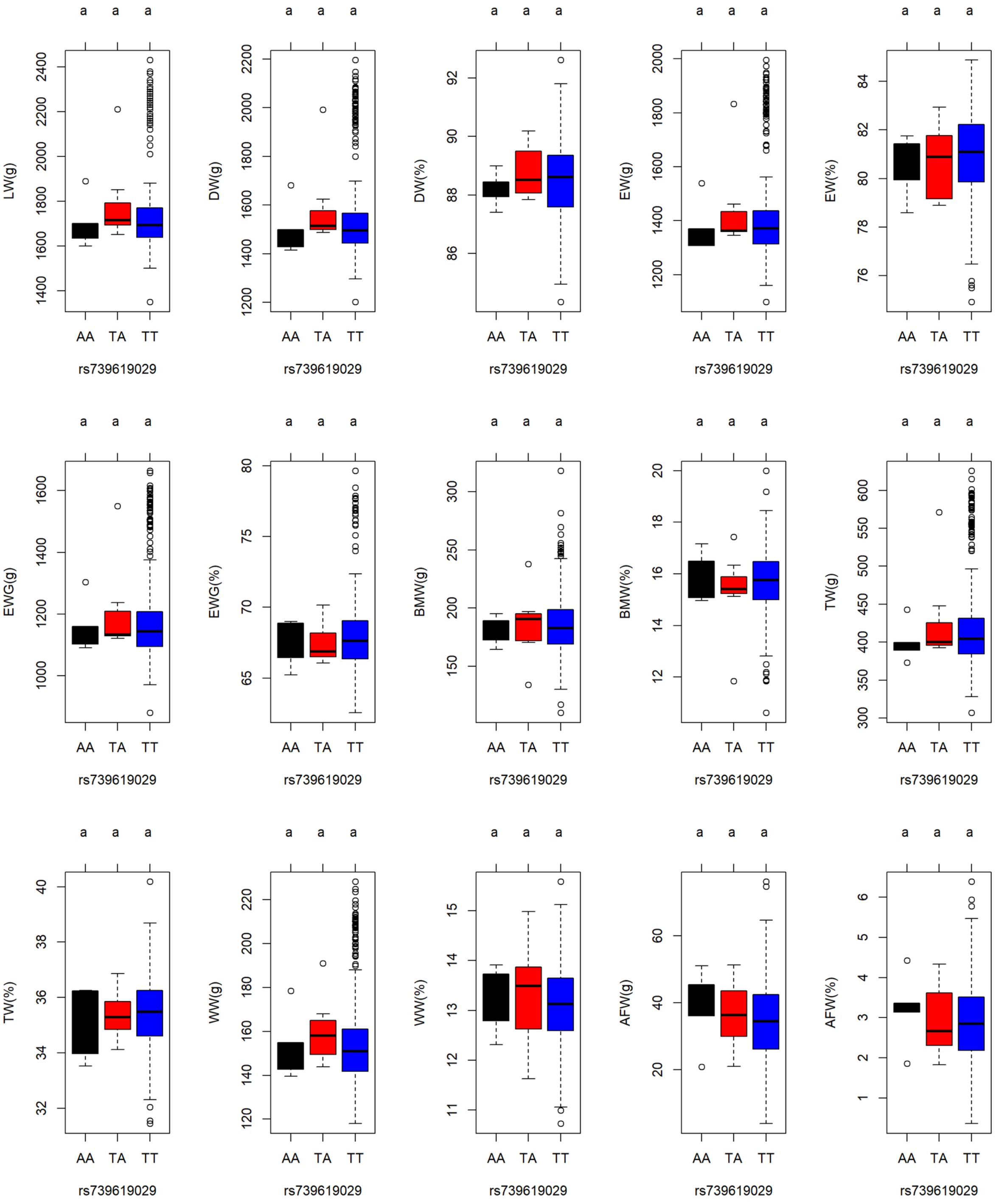

rs732603803

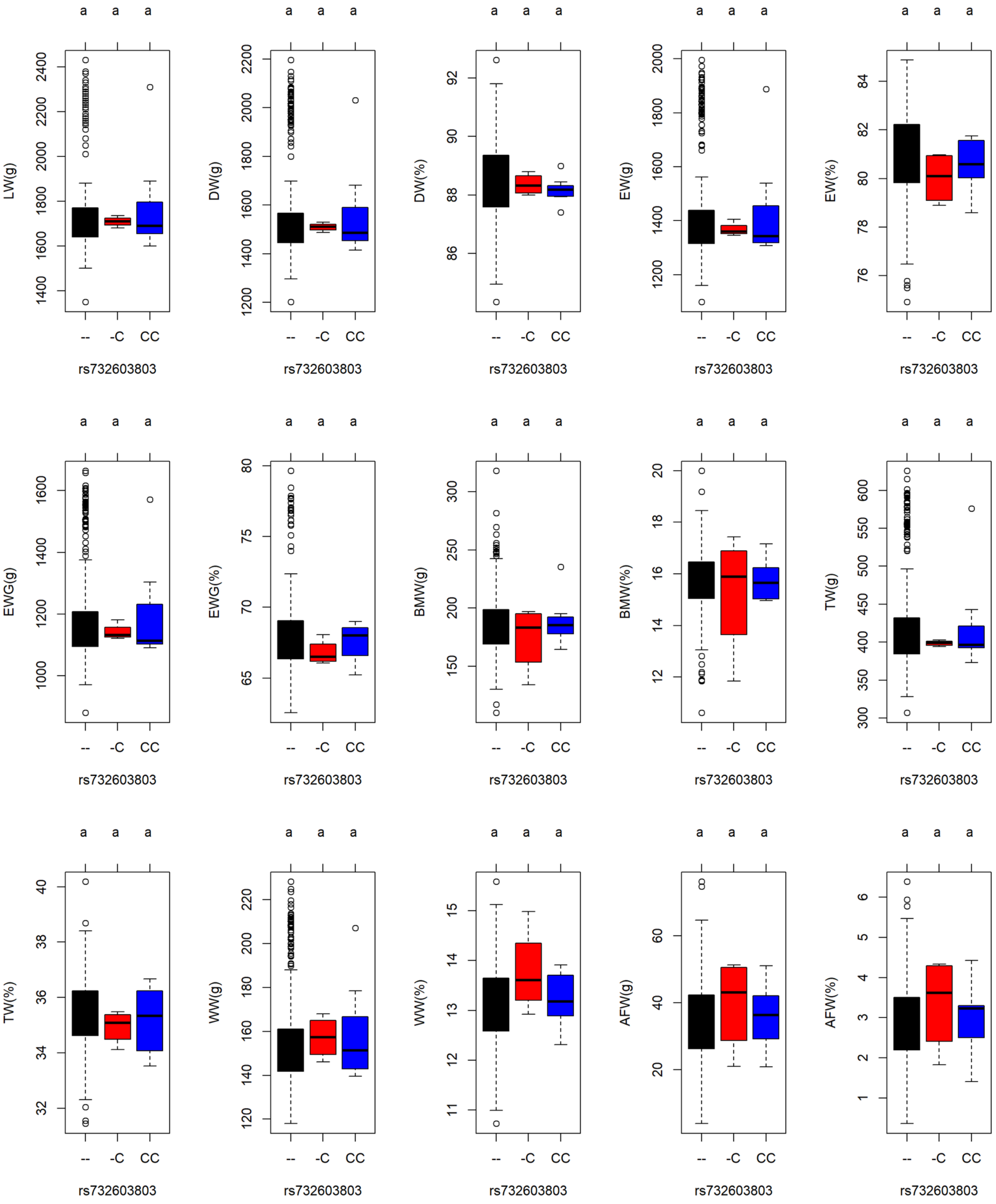

rs732200076

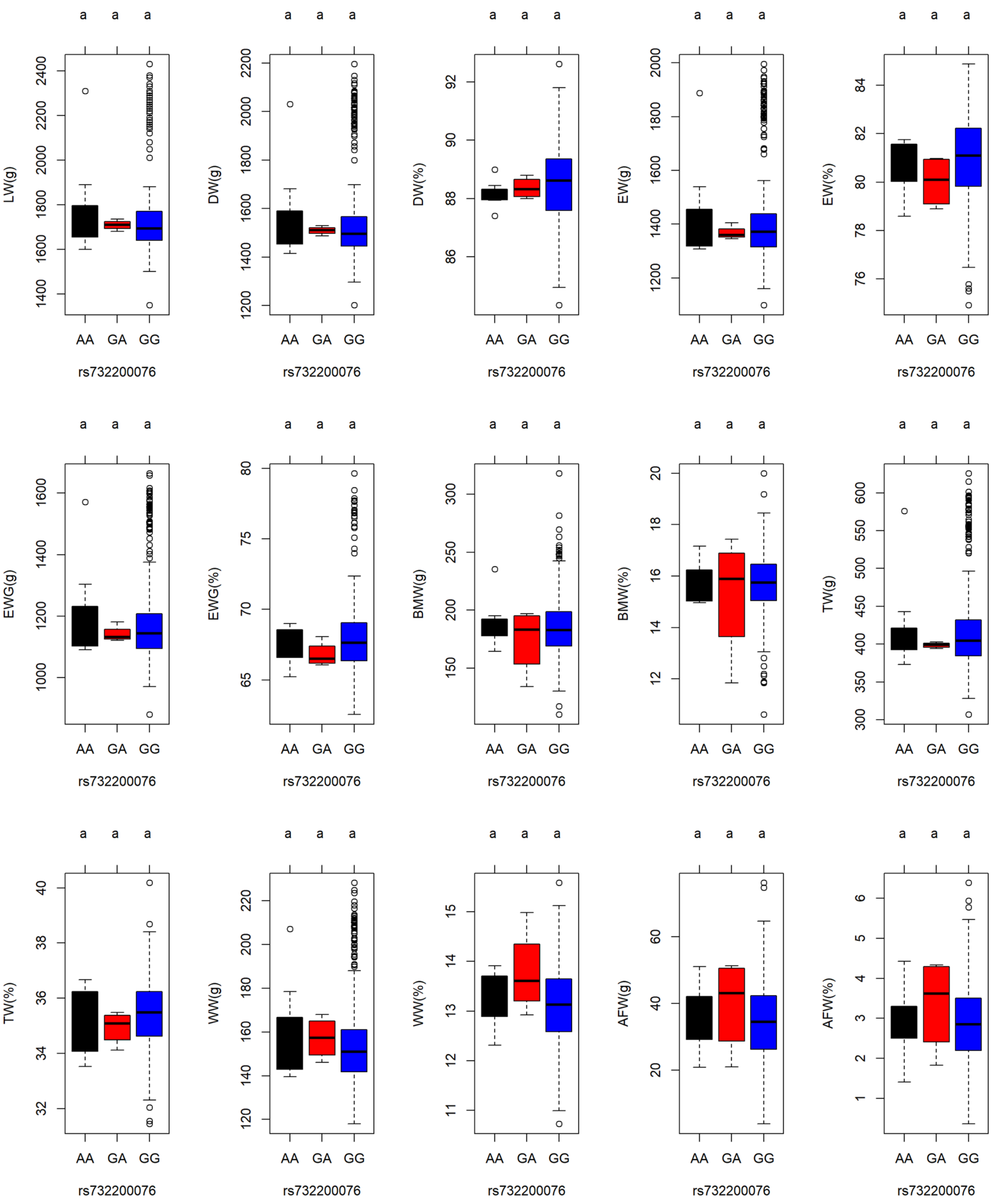

A56907254G

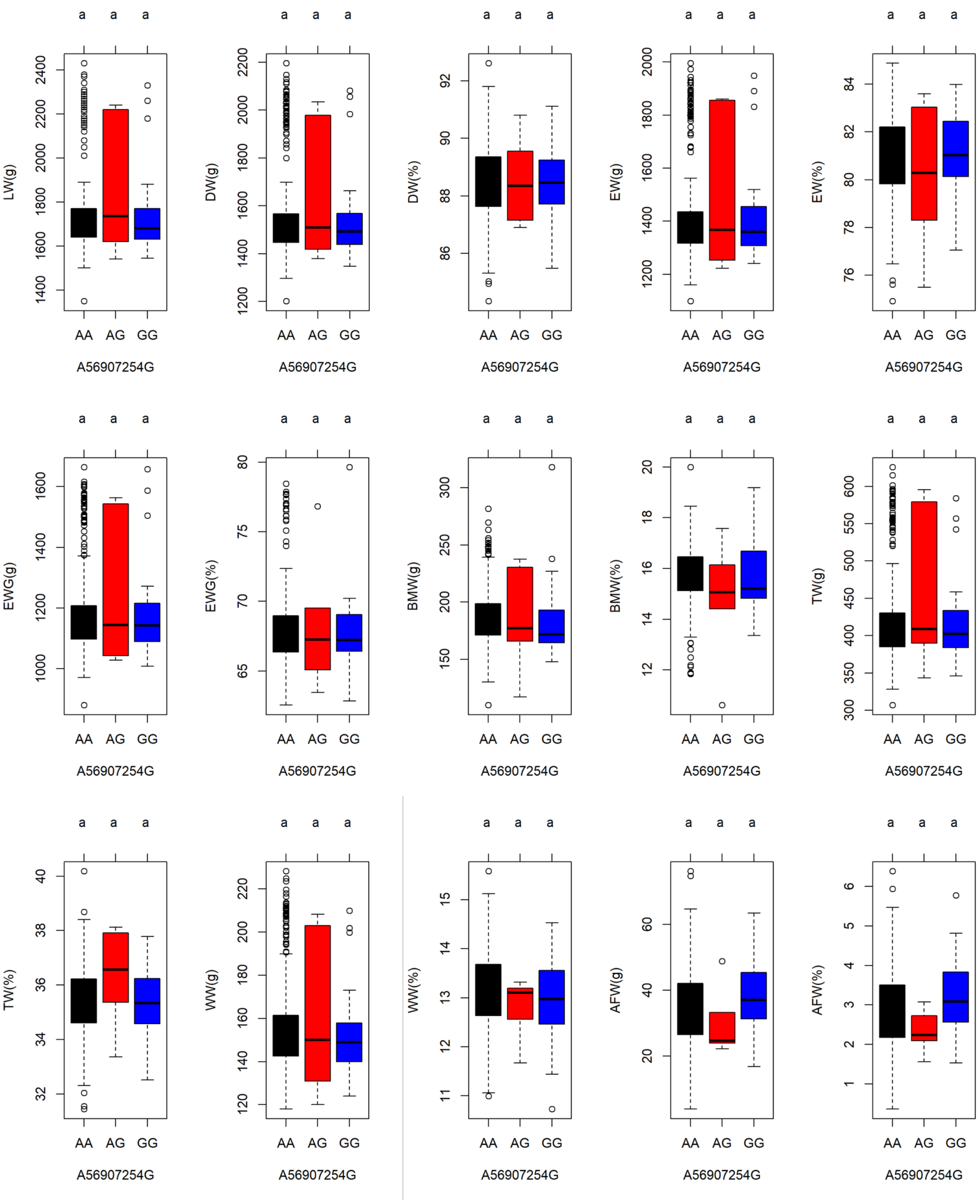
LW(g)
DW(g)
DW(%)
EW(g)
EW(%)
EWG(g)
EWG(%)
BMW(g)
BMW(%)
TW(g)
TW(%)
WW(g)
WW(%)
AFW(g)
AFW(%)
a a a
AA AG GG
A56907254G

rs730937100

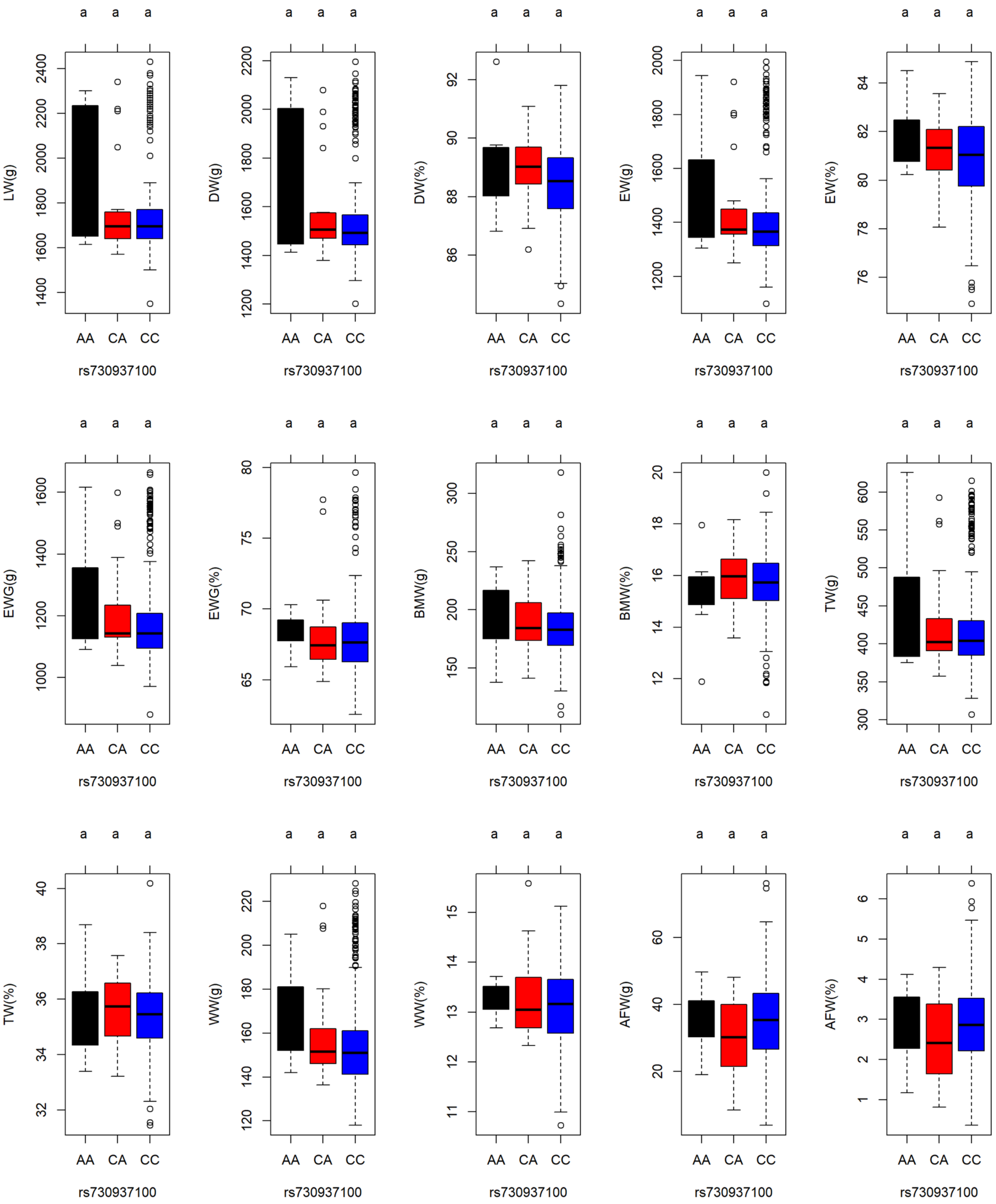

rs314708583

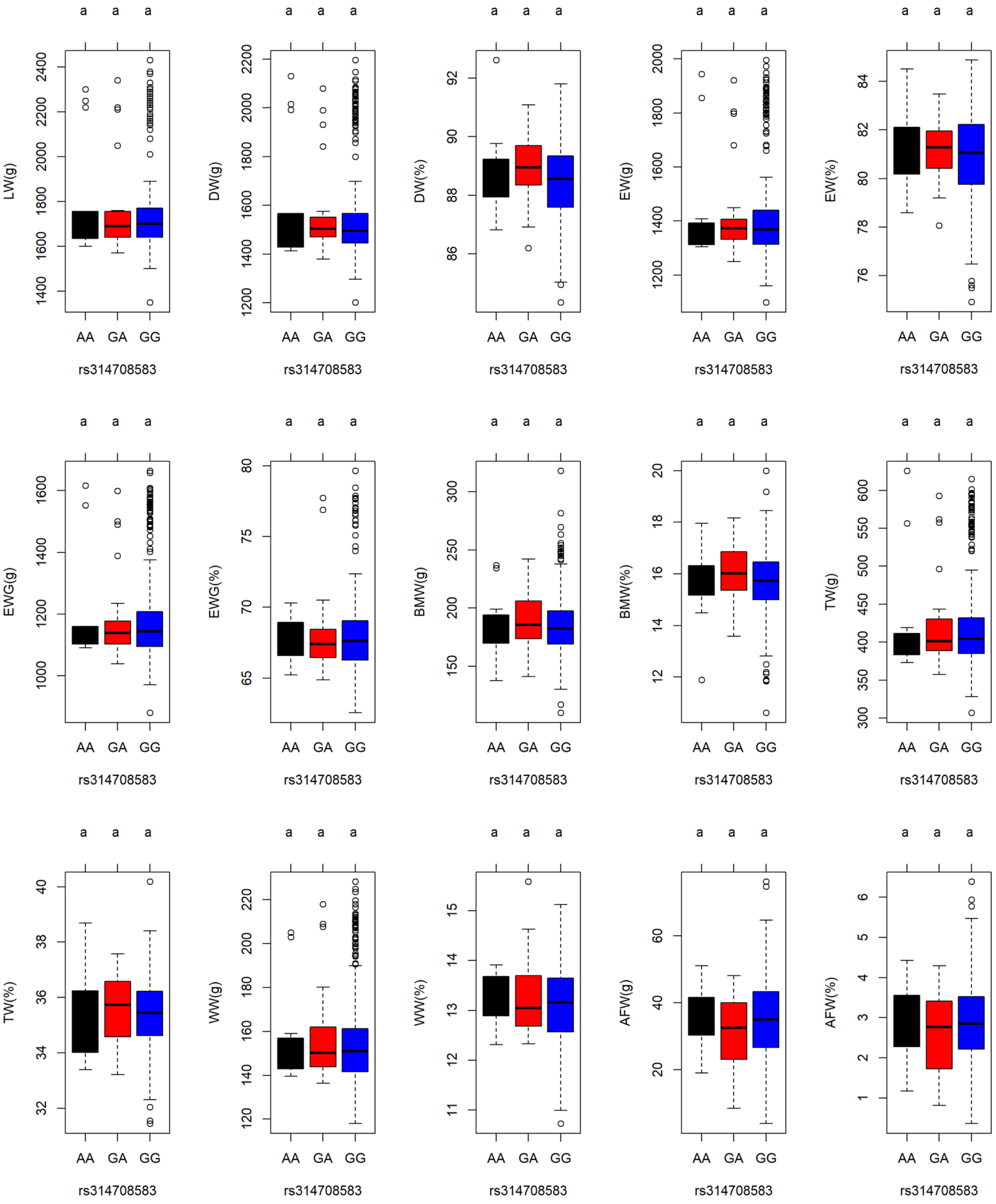

T56907387C

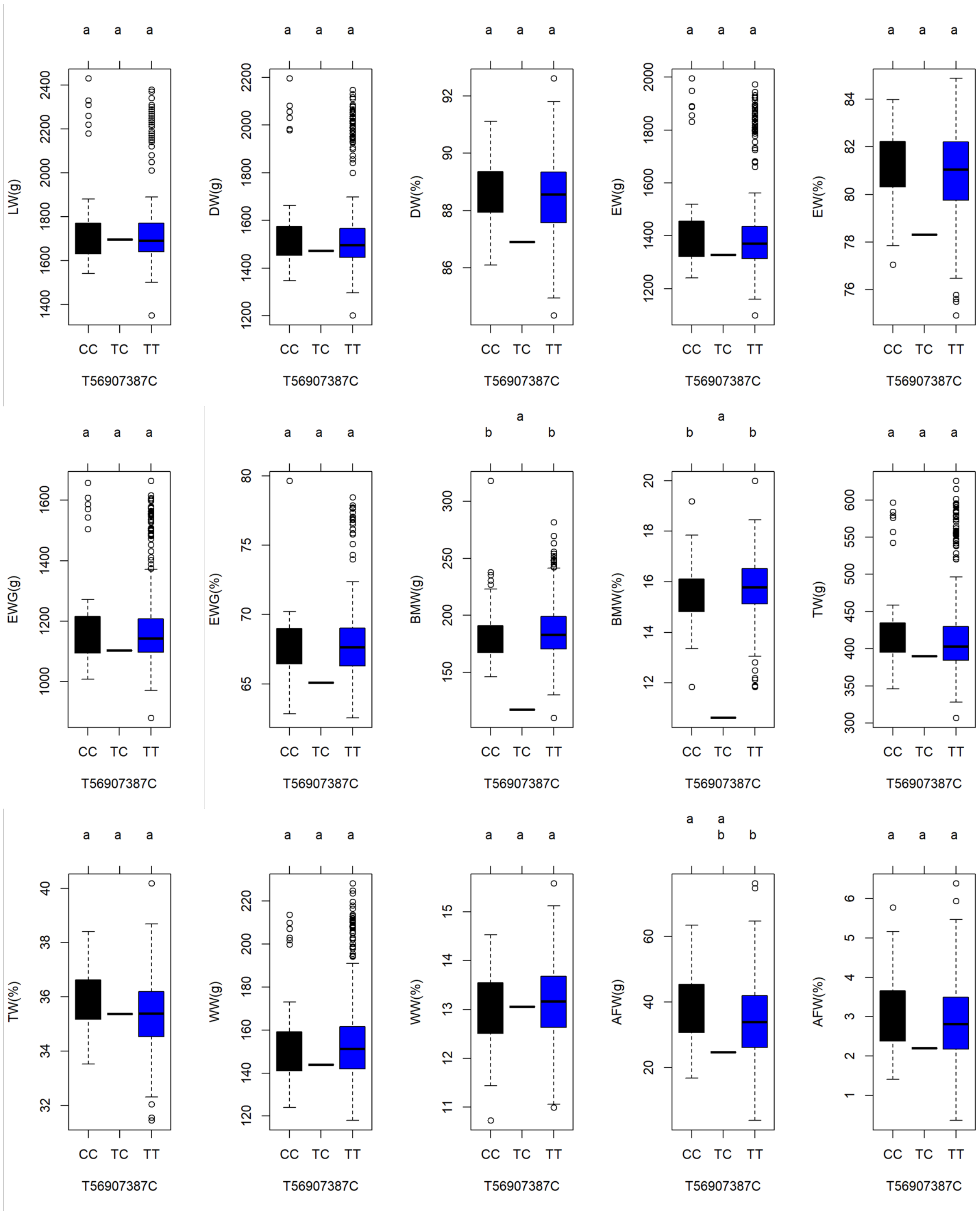
LW(g)
DW(g)
DW(%)
EW(g)
EW(%)
EWG(g)
EWG(%)
BMW(g)
BMW(%)
TW(g)
TW(%)
WW(g)
WW(%)
AFW(g)
AFW(%)
CC TC TT
T56907387C

rs735640639

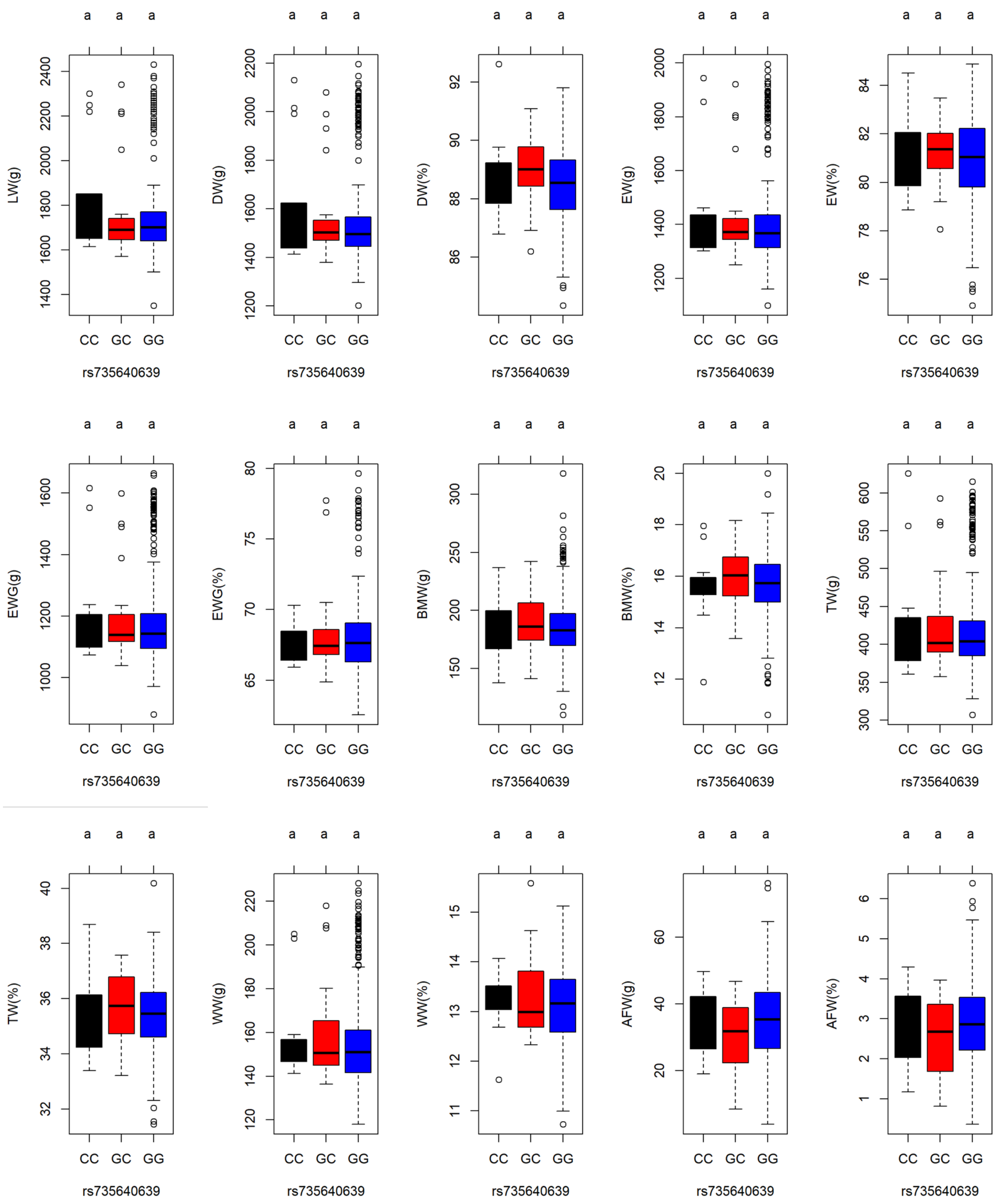

rs739066967

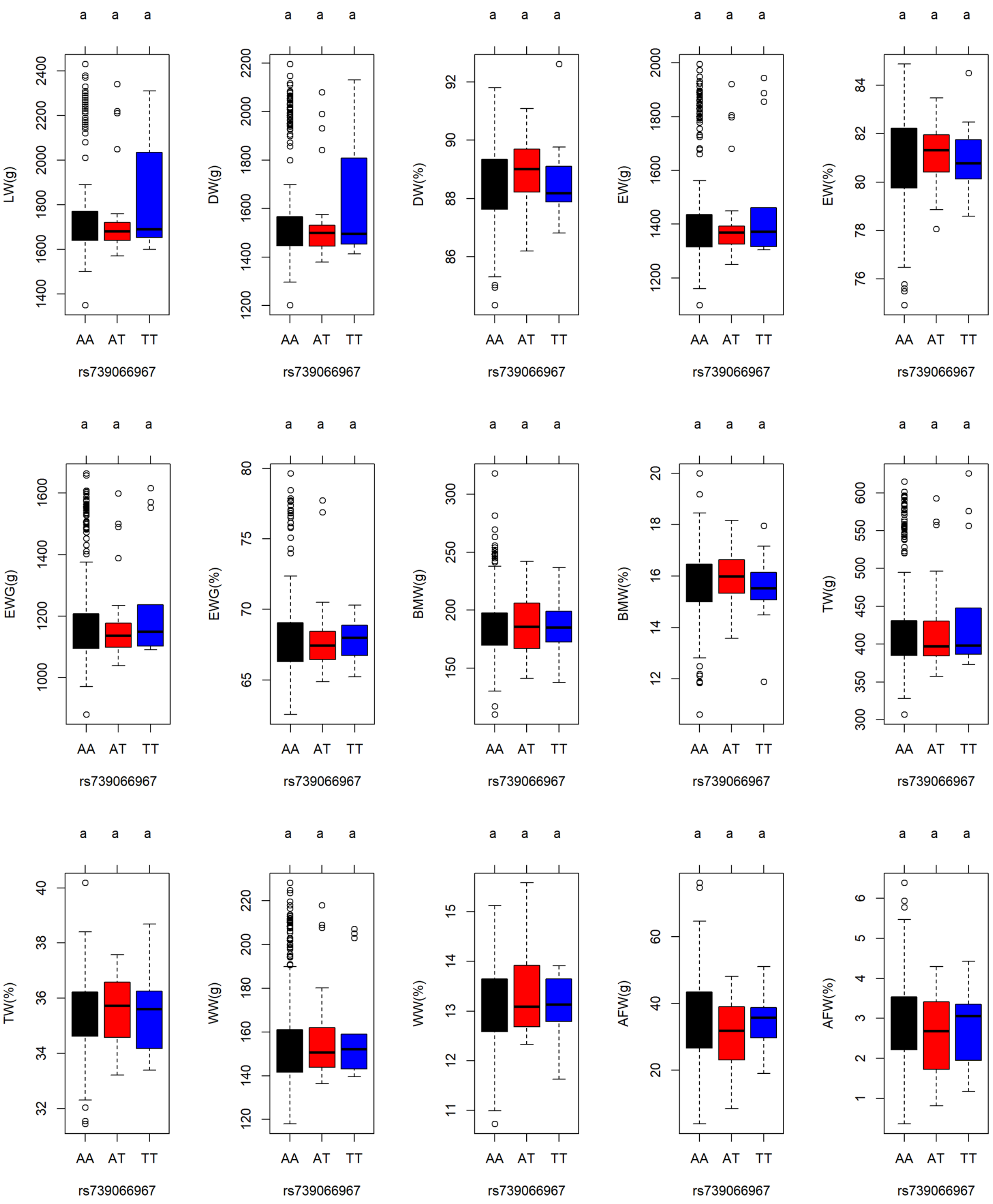

rs731721017

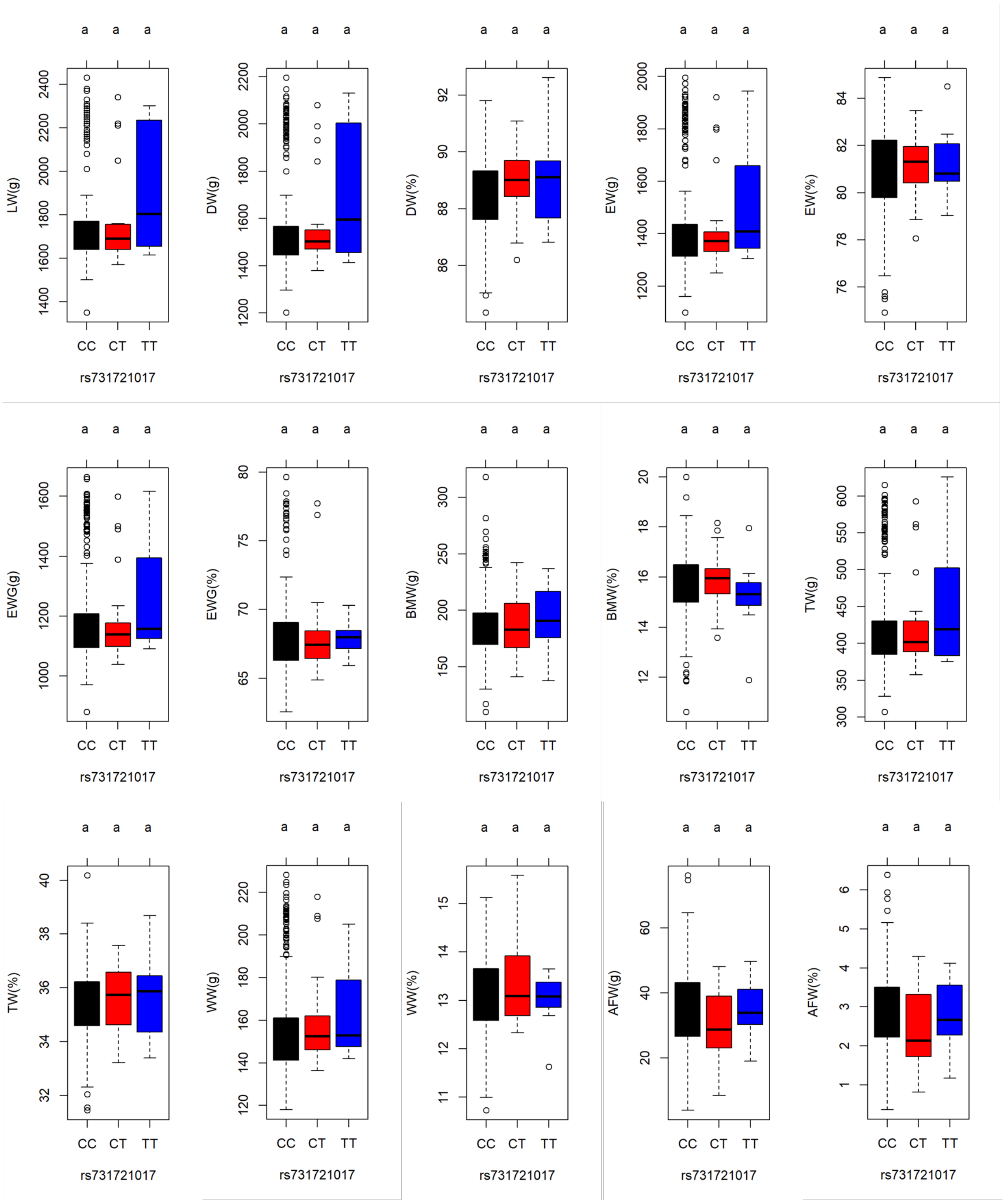

rs735069986

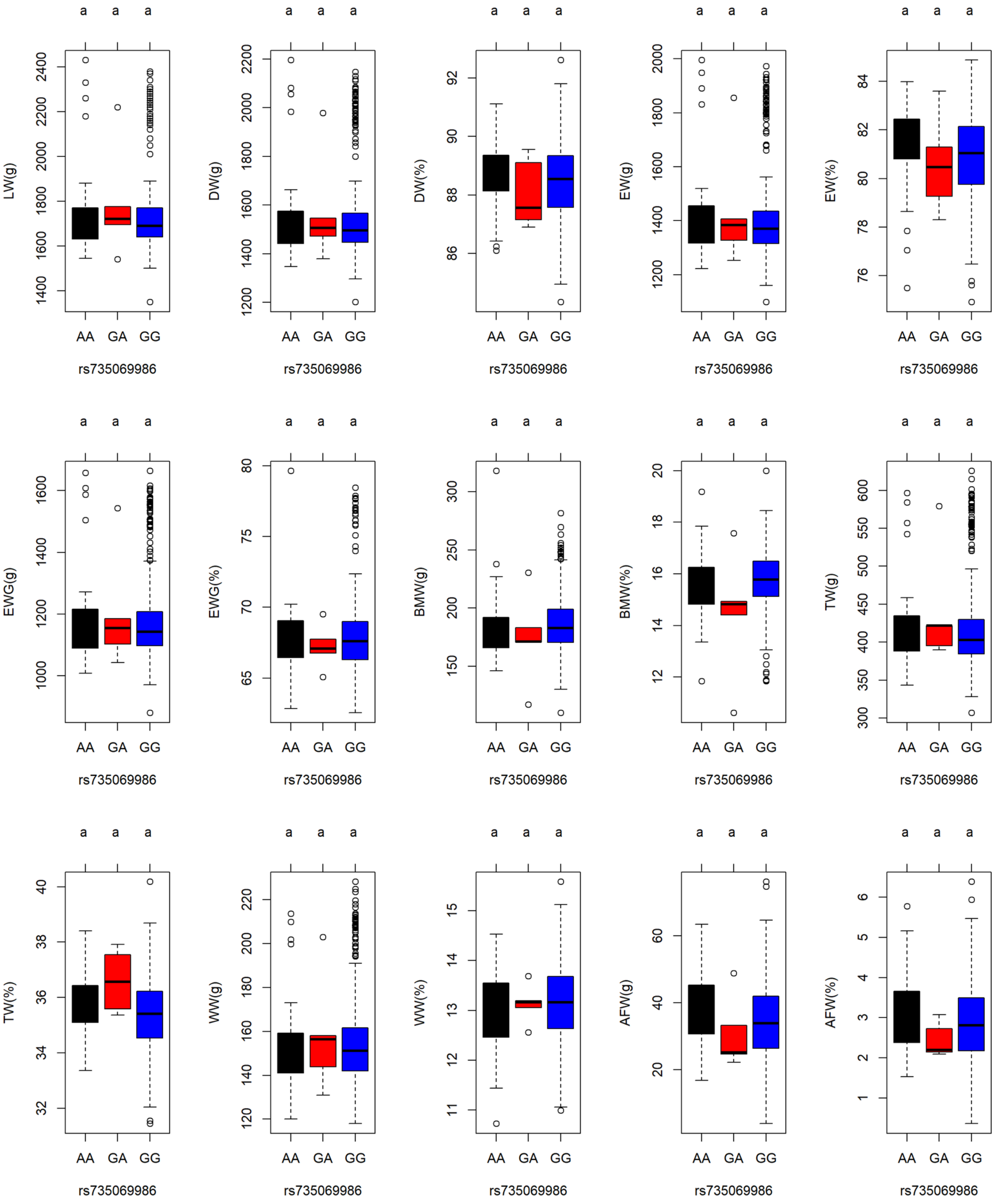

rs732782882

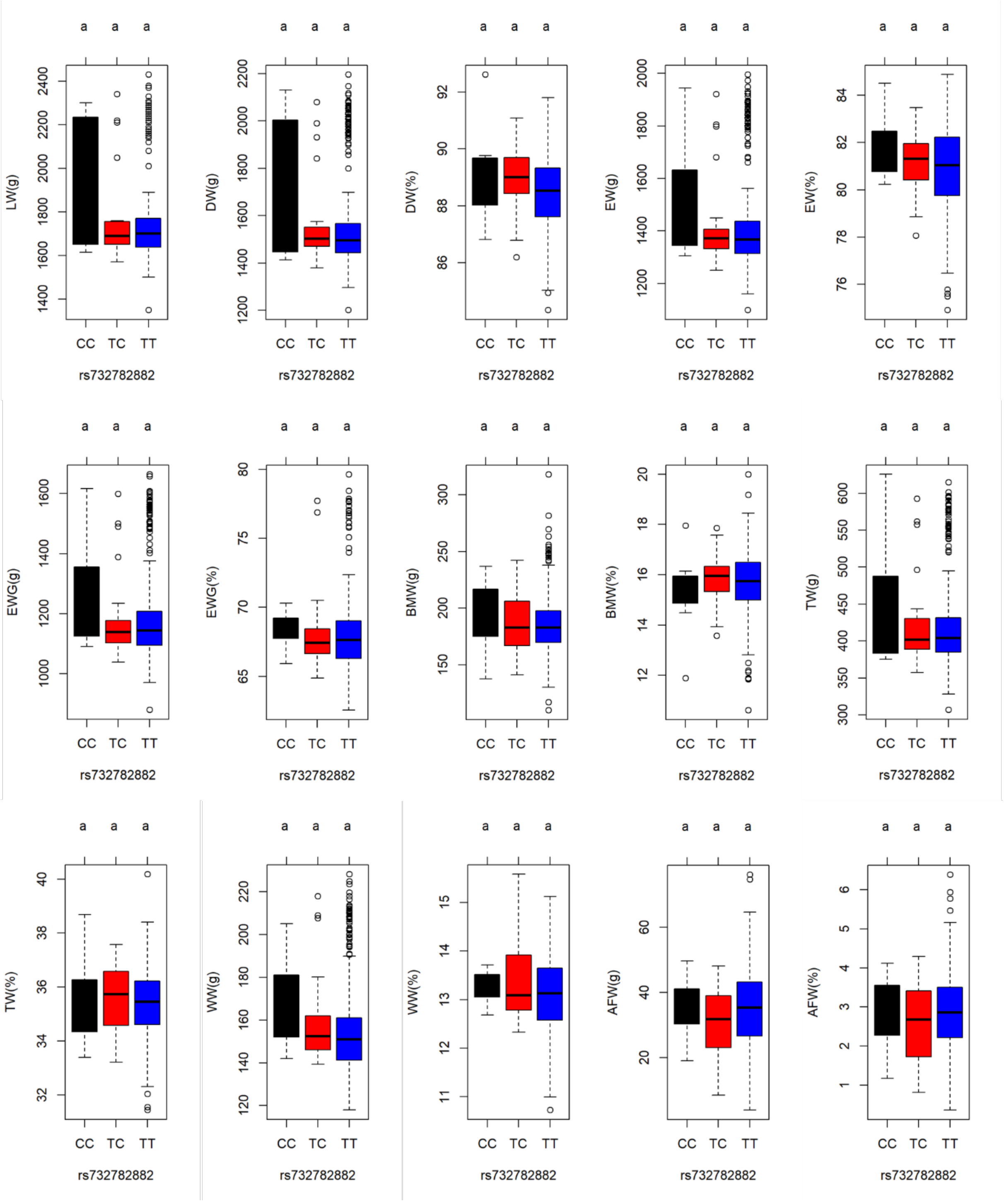

rs734452255

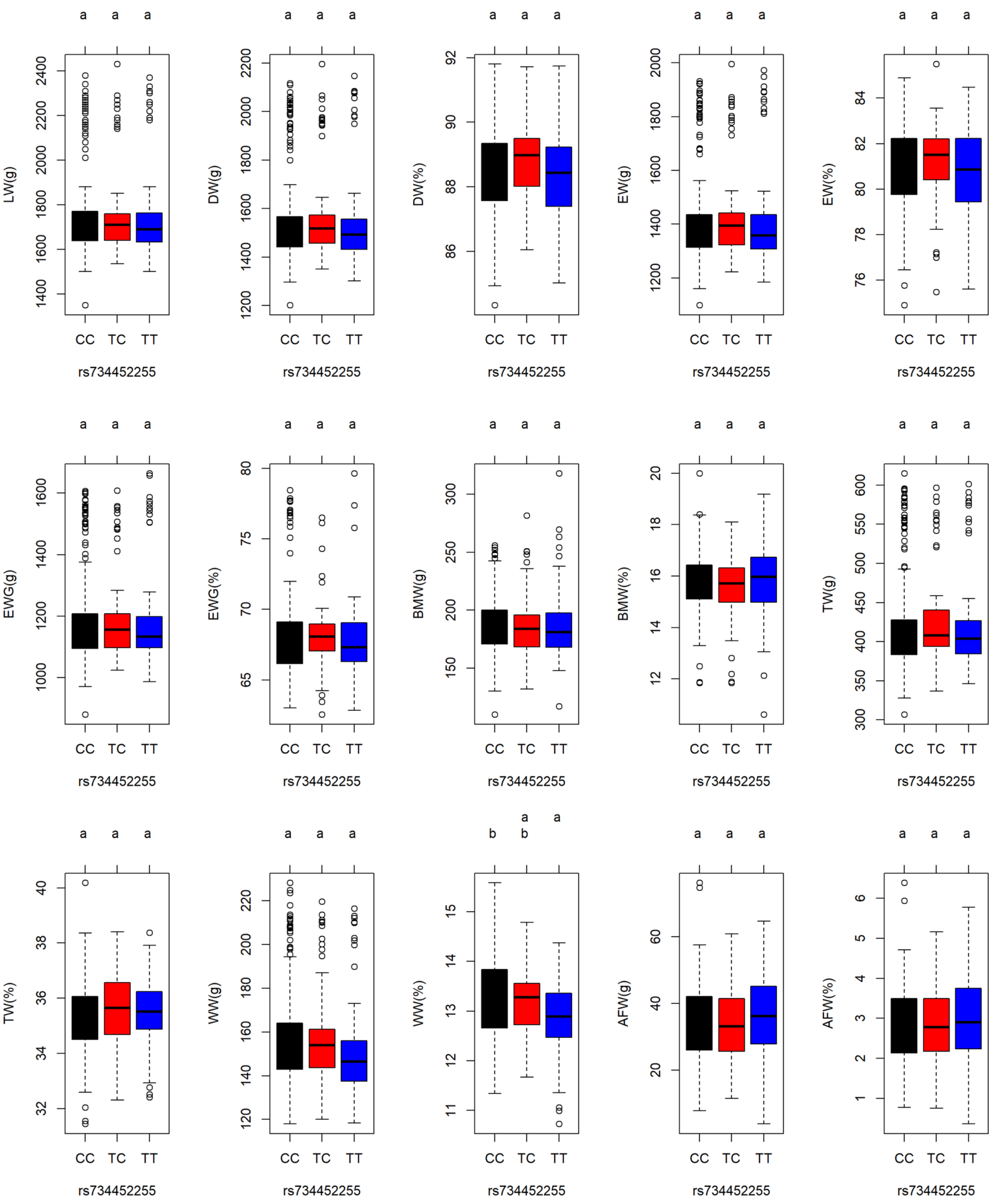

rs736763713

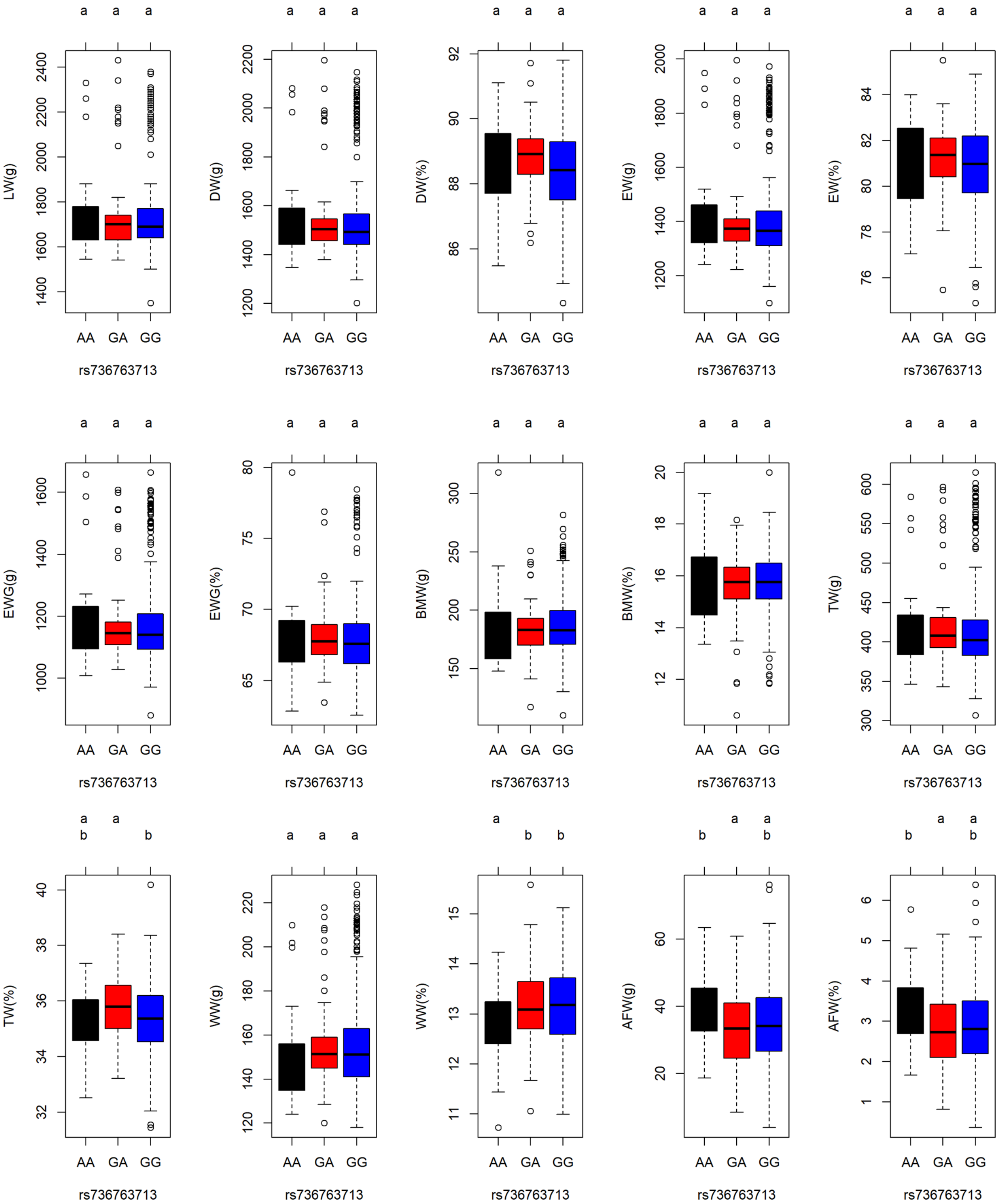

rs741366906

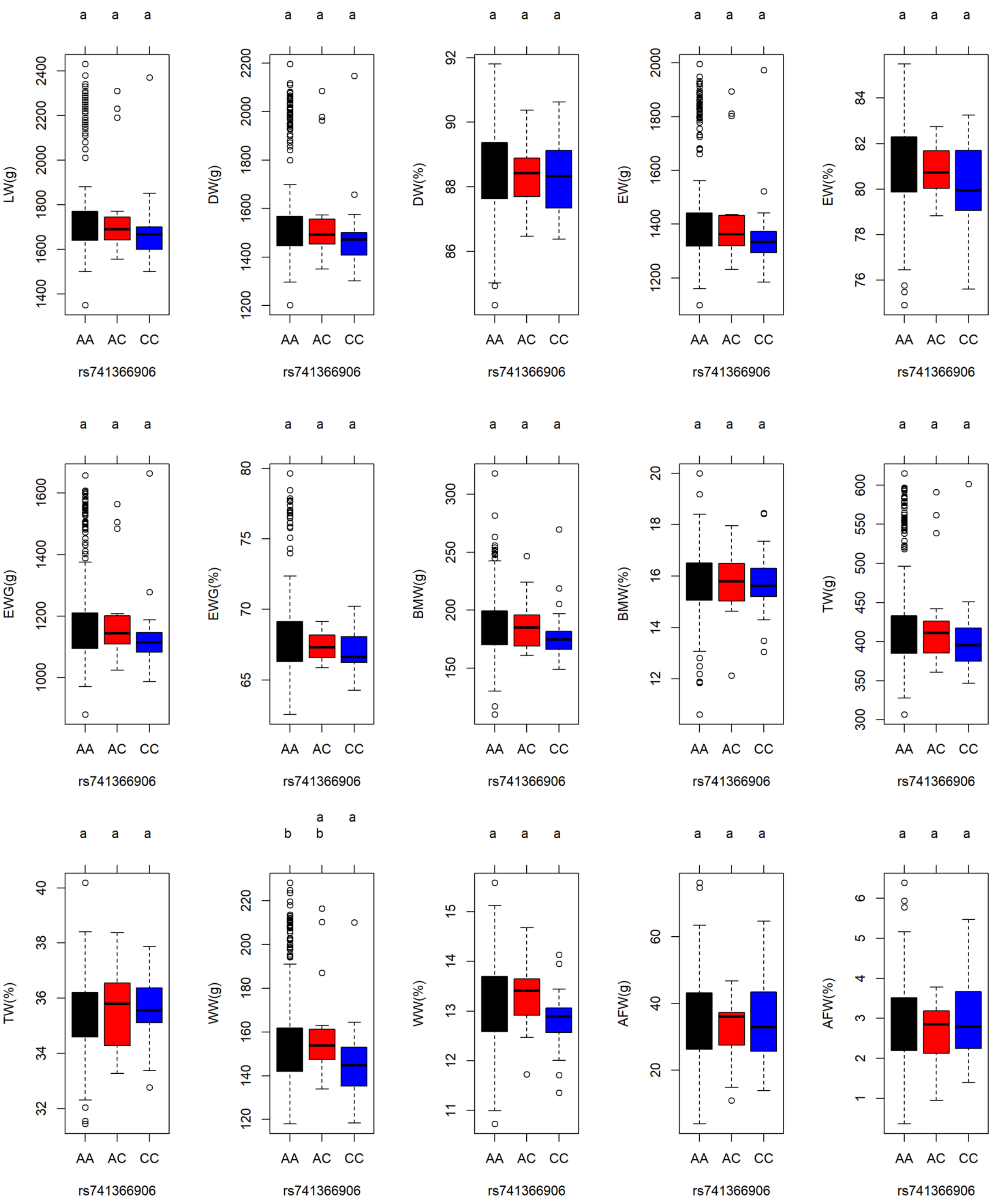

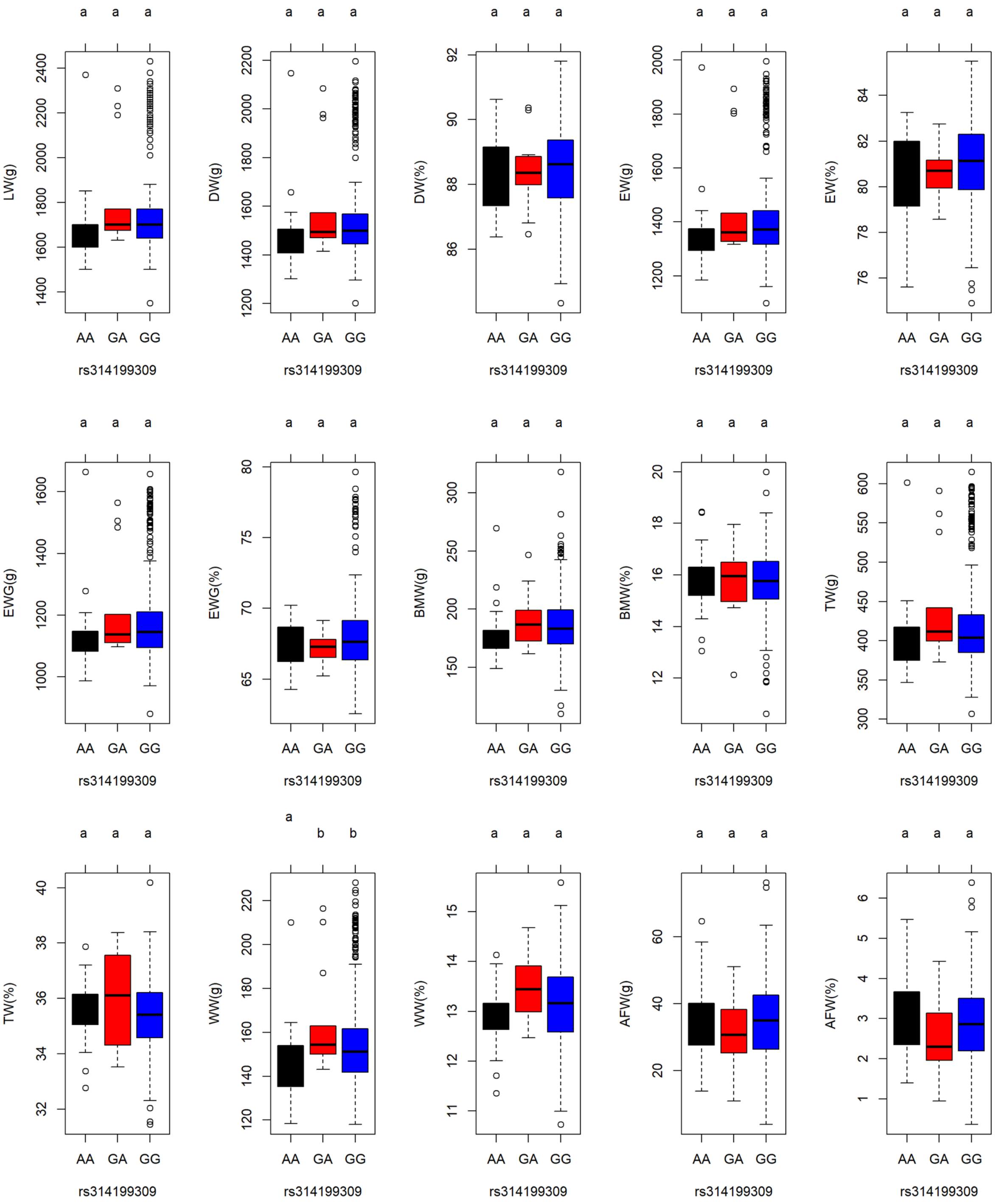

rs314199309

BLOCK1

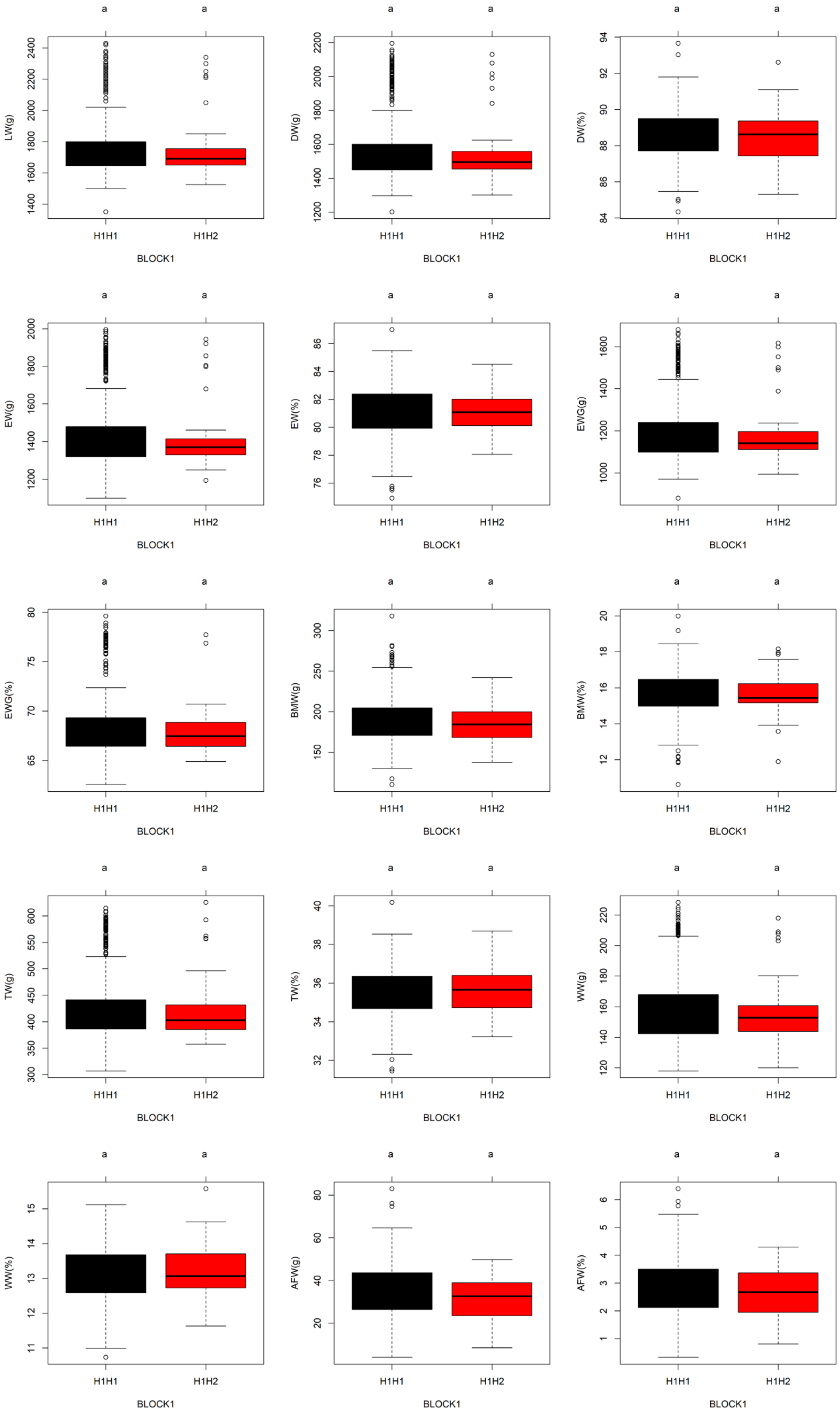
LW(g)
DW(g)
DW(%)
EW(g)
EW(%)
EWG(g)
EWG(%)
BMW(g)
BMW(%)
TW(g)
TW(%)
WW(g)
WW(%)
AFW(g)
AFW(%)
a
a
H1H1
H1H2
BLOCK1

BLOCK2

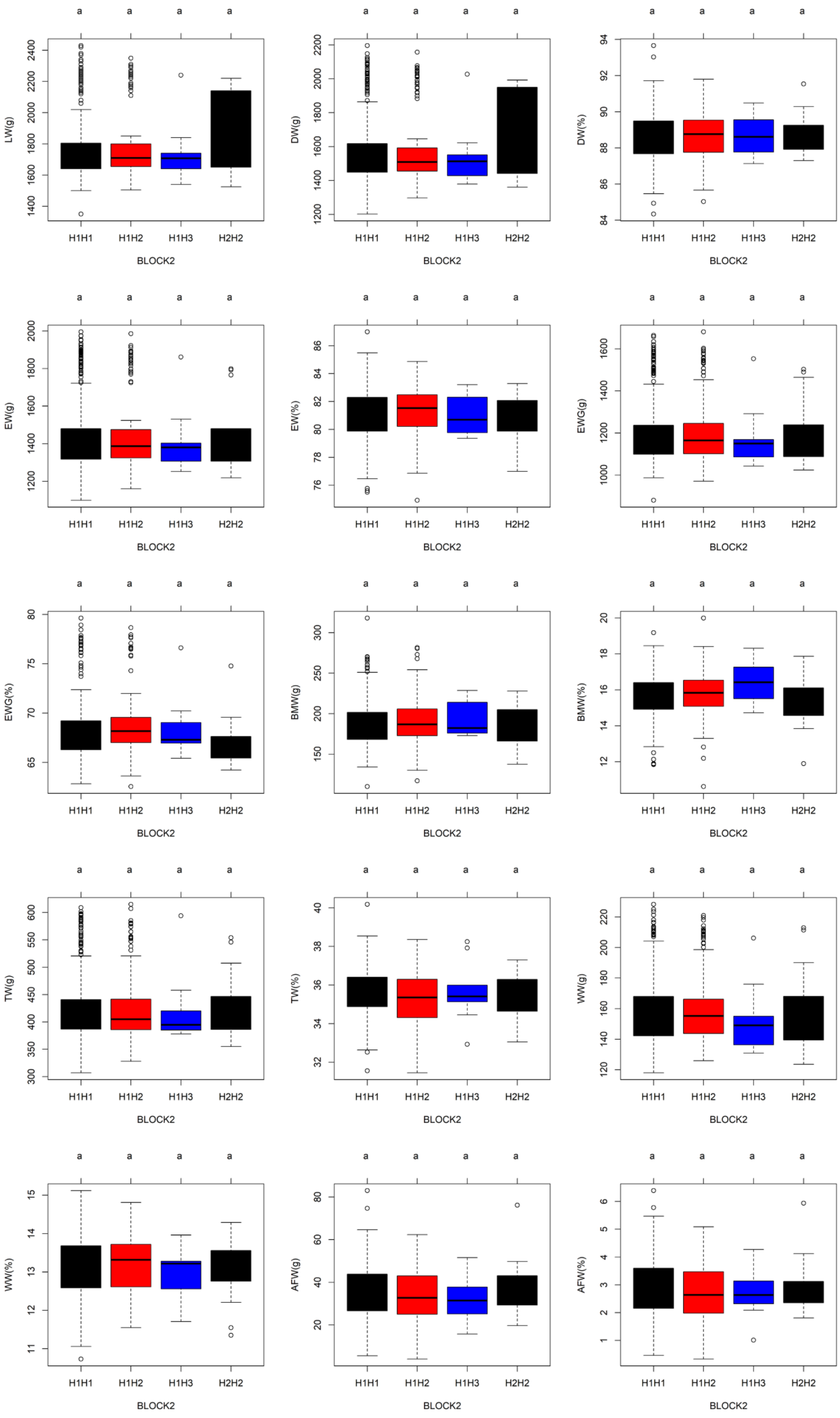
LW(g)
DW(g)
DW(%)
EW(g)
EW(%)
EWG(g)
EWG(%)
BMW(g)
BMW(%)
TW(g)
TW(%)
WW(g)
WW(%)
AFW(g)
AFW(%)
H1H1
H1H2
H1H3
H2H2
BLOCK2

BLOCK3

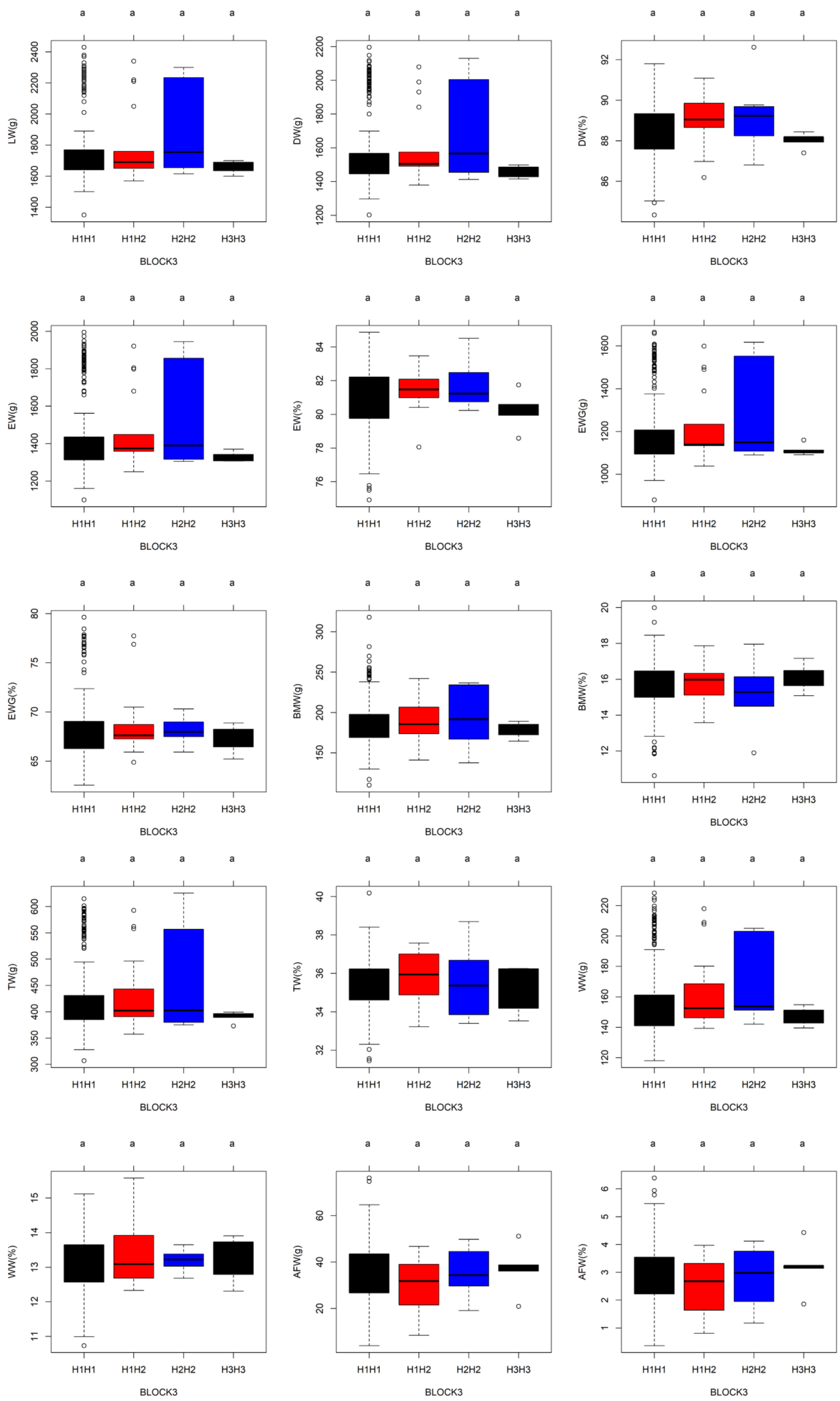

LW(g)
DW(g)
DW(%)
EW(g)
EW(%)
EWG(g)
EWG(%)
BMW(g)
BMW(%)
TW(g)
TW(%)
WW(g)
WW(%)
AFW(g)
AFW(%)
H1H1
H1H2
H2H2
H3H3
BLOCK3
a

SUPPLEMENTARY TABLES

Table S1. Association between SNPs and carcass traits

SNPs	LW	DW	DWP	EW	EWP	EWG	EWGP	BMW	BMWP	TW	TWP	WW	WWP	AFW	AFWP
rs16283693	0.1788	2.89E-06	2.56E-06	1.49E-05	0.000732	7.46E-07	0.001266	1.49E-05	0.00163	0.5855	0.0178	0.168	0.1419	1.45E-09	4.55E-08
rs16283694	0.7667	3.80E-03	6.97E-04	2.29E-03	0.003434	2.20E-03	0.2854	3.07E-03	0.006568	0.6731	0.02454	0.6542	0.07542	6.81E-05	1.88E-03
rs313332806	0.345	1.21E-02	1.95E-02	3.89E-02	0.1906	4.85E-03	0.2477	8.52E-04	0.2206	0.5188	0.1092	0.3302	0.298	3.25E-03	4.08E-03
rs16283697	0.659	7.54E-01	6.07E-01	4.96E-01	0.9833	4.51E-01	0.3934	4.33E-01	0.198	0.9962	0.1359	0.6225	0.07656	5.16E-01	2.94E-01
rs738527022	0.9285	4.79E-02	2.41E-03	1.61E-01	0.0881	7.29E-02	0.5444	1.19E-01	0.01695	0.8273	0.02781	0.9116	0.0455	1.91E-02	1.26E-01
rs315414930	0.8317	1.91E-02	5.13E-04	1.47E-01	0.06679	1.15E-01	0.1784	1.09E-01	0.01663	0.3982	0.07056	0.7467	0.1022	1.08E-02	8.64E-02
rs732101949	0.4264	4.33E-02	5.53E-04	1.87E-01	0.04733	1.54E-01	0.2955	3.30E-01	0.03085	0.502	0.07245	0.4481	0.09015	1.06E-02	1.63E-01
rs735639580	0.5651	3.01E-02	3.33E-04	1.57E-01	0.04268	1.53E-01	0.3011	2.20E-01	0.0213	0.5609	0.06598	0.5703	0.0843	1.31E-02	1.42E-01
C56889810T	0.877	8.33E-01	7.41E-01	6.08E-01	0.4998	8.16E-01	0.2373	7.39E-01	0.4196	0.8193	0.1614	0.8033	0.1277	9.03E-01	8.40E-01
rs1059454729	0.81	8.89E-01	8.44E-01	6.26E-01	0.5348	8.41E-01	0.2928	8.38E-01	0.3726	0.6588	0.1116	0.7157	0.08315	9.06E-01	8.46E-01
rs317230140	0.4869	1.78E-02	3.88E-03	1.22E-01	0.1819	1.38E-02	0.08735	5.96E-03	0.006957	0.5398	0.05395	0.5602	0.1048	1.27E-02	3.70E-02
C56889913G	0.7591	8.06E-01	5.45E-01	1.53E-01	0.299	6.76E-01	0.9107	4.79E-01	0.3512	0.7885	0.4083	0.6004	0.3	4.84E-01	3.56E-01
T56890010C	0.6974	7.63E-01	5.64E-01	1.47E-01	0.301	7.00E-01	0.7707	3.68E-01	0.2874	0.7467	0.3418	0.588	0.2519	5.15E-01	3.54E-01
G56890028A	0.1905	5.08E-01	8.12E-01	5.29E-01	0.9416	2.68E-01	0.7843	5.01E-01	0.8537	0.4858	0.9212	0.2883	0.9928	7.66E-01	2.96E-01
rs731237306	0.7086	8.24E-01	4.75E-01	2.30E-01	0.4727	9.13E-01	0.4946	2.48E-01	0.5261	0.7516	0.637	0.5542	0.4348	4.52E-01	3.19E-01
C56890154T	0.8418	8.58E-01	4.99E-01	1.73E-01	0.3271	7.65E-01	0.6055	2.61E-01	0.5035	0.6778	0.2466	0.6279	0.1497	4.49E-01	3.51E-01
rs16283703	0.3215	7.36E-01	4.56E-01	2.73E-01	0.01963	8.95E-01	0.7342	6.64E-02	0.6577	0.3284	0.4466	0.3496	0.3735	6.14E-01	7.07E-01
rs317648691	0.7433	4.53E-02	5.48E-03	1.47E-01	0.1213	3.57E-02	0.1483	8.60E-02	0.01047	0.7838	0.04299	0.8787	0.06921	2.16E-02	8.35E-02
rs315560057	0.7433	4.53E-02	5.48E-03	1.47E-01	0.1213	3.57E-02	0.1483	8.60E-02	0.01047	0.7838	0.04299	0.8787	0.06921	2.16E-02	8.35E-02
A56906727T	0.186	7.24E-01	5.95E-01	3.76E-01	0.7112	8.78E-01	0.4888	6.57E-01	0.5188	0.4306	0.5109	0.3351	0.5857	8.81E-01	3.90E-01
rs314492304	0.2778	1.54E-01	2.73E-01	2.35E-01	0.01313	4.96E-01	0.2737	5.17E-01	0.6779	0.2766	0.1369	0.2411	0.2336	5.33E-01	2.36E-01

rs734035893	0.2293	2.61E-02	2.49E-01	1.30E-01	0.899	1.44E-02	0.05227	1.58E-01	0.1419	0.3003	0.7833	0.01502	0.4288	7.32E-02	9.14E-02
rs738952872	0.308	7.30E-01	7.71E-01	3.89E-01	0.6142	9.97E-01	0.2153	9.39E-01	0.7526	0.3284	0.4274	0.3337	0.5055	8.80E-01	5.26E-01
rs739619029	0.308	7.30E-01	7.71E-01	3.89E-01	0.6142	9.97E-01	0.2153	9.39E-01	0.7526	0.3284	0.4274	0.3337	0.5055	8.80E-01	5.26E-01
rs732603803	0.2693	7.65E-01	7.67E-01	2.97E-01	0.4695	9.38E-01	0.9085	7.37E-01	0.5472	0.6345	0.5584	0.4223	0.5756	8.63E-01	5.33E-01
rs732200076	0.2693	7.65E-01	7.67E-01	2.97E-01	0.4695	9.38E-01	0.9085	7.37E-01	0.5472	0.6345	0.5584	0.4223	0.5756	8.63E-01	5.33E-01
A56907254G	0.3967	4.29E-01	6.28E-01	6.66E-01	0.2686	2.08E-01	0.7247	8.23E-01	0.5211	0.7068	0.09474	0.3503	0.1151	6.14E-01	4.78E-01
rs730937100	0.3635	1.44E-04	1.18E-05	1.17E-03	0.005454	2.72E-04	0.01339	4.76E-05	0.12	0.7811	0.131	0.6628	0.4988	1.93E-05	5.12E-05
rs314708583	0.214	2.50E-04	1.33E-04	2.14E-03	0.02044	8.82E-04	0.007497	2.17E-05	0.05218	0.4749	0.358	0.351	0.9479	1.05E-04	1.14E-04
T56907387C	0.142	5.36E-02	1.06E-01	8.68E-01	0.3993	2.88E-02	0.6625	6.93E-01	0.4924	0.7295	0.01689	0.08332	0.0318	3.59E-01	2.24E-01
rs735640639	0.6428	8.34E-04	1.25E-04	2.95E-03	0.01109	5.08E-04	0.0609	1.52E-04	0.1286	0.9064	0.1956	0.8333	0.583	5.29E-05	3.74E-04
rs739066967	0.4994	1.45E-03	3.82E-04	3.55E-03	0.0146	1.87E-03	0.0188	1.37E-05	0.05592	0.7031	0.2689	0.5557	0.7518	1.67E-04	7.21E-04
rs731721017	0.5627	1.94E-04	2.44E-05	2.84E-04	0.001427	1.46E-04	0.006191	5.71E-05	0.1117	0.9366	0.1197	0.8069	0.4655	3.16E-06	3.86E-05
rs735069986	0.229	1.10E-01	2.39E-01	9.38E-01	0.2722	8.93E-02	0.9629	5.53E-01	0.5656	0.6544	0.04141	0.1031	0.0792	3.70E-01	2.65E-01
rs732782882	0.2536	4.76E-05	7.02E-06	9.58E-04	0.009303	1.22E-04	0.01004	2.18E-05	0.03775	0.9771	0.1804	0.4792	0.6571	1.08E-05	1.72E-05
rs734452255	0.2638	7.06E-02	1.06E-01	4.37E-02	0.000126	7.25E-01	0.6321	1.19E-01	0.2004	0.1271	0.3105	0.2339	0.4258	9.55E-01	2.18E-01
rs736763713	0.4637	1.02E-01	1.27E-01	4.50E-01	0.05721	1.70E-01	0.8357	4.78E-01	0.5703	0.2349	0.1435	0.2266	0.2053	3.95E-02	2.50E-01
rs741366906	0.4783	8.88E-01	1.68E-01	7.87E-02	0.1563	1.94E-01	0.9381	8.05E-01	0.8061	0.2547	0.7185	0.41	0.8046	1.53E-01	3.47E-01
rs314199309	0.5207	9.83E-01	1.28E-01	1.41E-01	0.2658	1.84E-01	0.8321	8.25E-01	0.6269	0.244	0.8407	0.4891	0.9339	1.58E-01	3.79E-01

Table S2 Association between BLOCKs and carcass traits

	block1	block2	block3
LW	0.2453	0.9368	0.2866
DW	0.2246	0.8853	0.5559
DWP	0.6752	0.8027	0.167
EW	0.2676	0.03	4.24E-06
EWP	0.9072	0.008559	2.14E-06
EWG	0.3103	0.05781	6.02E-06
EWGP	0.5971	0.0286	1.62E-06
BMW	0.5198	0.2629	0.00071
BMWP	0.9999	0.1832	0.001444
TW	0.6403	0.01029	3.09E-05
TWP	0.4383	2.88E-04	4.43E-06
WW	0.8572	0.1182	2.23E-04
WWP	0.4039	0.07091	0.002136
AFW	0.1296	0.1238	0.1573
AFWP	0.1978	0.1034	0.2994

Table S3. MEAN ± SD of traits by diplotypes of BLOCKs

		BLOCK1		BLOCK2				BLOCK3			
Traits	statistics	H1H1	H1H2	H1H1	H1H2	H1H3	H2H2	H1H1	H1H2	H2H2	H3H3
LW	mean	1795.74	1768.194	1794.104	1801.389	1739	1831.071	1756.455	1797.059	1921.429	1660
	sd	237.7067	219.7699	239.9079	239.7778	195.289	243.2487	206.0407	243.1246	317.6045	41.68333
DW	mean	1593.239	1566.639	1591.508	1598.611	1542.8	1627.857	1555.316	1600.706	1718.286	1461.2
	sd	223.5452	207.9339	225.4048	225.8653	186.0477	229.7798	193.1078	214.5981	314.0699	36.96214
DWP	mean	88.64896	88.53111	88.63267	88.6663	88.663	88.82143	88.49492	89.09235	89.21429	88.026
	sd	1.37062	1.464938	1.407594	1.329816	1.138264	1.202228	1.301286	1.213824	1.861575	0.393675
EW	mean	1457.631	1433.528	1455.263	1465.435	1411	1451.231	1421.952	1464.294	1533.5	1331
	sd	210.6554	192.0153	211.6823	212.4131	176.4351	203.8546	181.5863	201.2613	287.7038	26.24881
EWP	mean	81.07062	81.00306	81.01629	81.25157	81.057	80.44692	80.88514	81.47118	81.74167	80.196
	sd	1.854871	1.494664	1.877345	1.836044	1.38994	1.814187	1.796591	1.24511	1.578574	1.145962
EWG	mean	1215.66	1195.833	1212.204	1224.824	1177.6	1206.231	1186.093	1216.941	1277.667	1113.6
	sd	175.5576	160.1805	175.6796	178.5001	149.4361	169.8809	151.0113	169.1466	239.7404	27.07951
EWGP	mean	68.41286	68.09139	68.28221	68.78685	68.369	67.35462	67.92737	68.79529	68.11	67.102
	sd	3.241943	2.703324	3.250797	3.313957	3.203094	2.718255	2.742932	3.482928	1.464486	1.466244
BMW	mean	190.4957	187.2306	188.8971	193.0725	192.733	182.7392	186.0962	191.4612	193.1983	179.006
	sd	30.42951	25.92106	30.28866	32.55925	21.32645	26.23555	26.85019	28.27844	38.66808	10.21372
BMWP	mean	15.68218	15.69028	15.59563	15.75787	16.421	15.21154	15.70234	15.74882	15.17833	16.074
	sd	1.253584	1.286154	1.2291	1.335715	1.131454	1.606802	1.262818	1.202746	1.995519	0.79858
TW	mean	432.544	427.2525	432.4254	433.8094	420.408	426.1469	420.8918	436.9876	457.1383	390.78
	sd	71.17797	68.34896	70.43136	74.13956	65.3145	68.10184	61.60631	70.89134	107.2053	10.52551
TWP	mean	35.50868	35.64306	35.60842	35.32361	35.634	35.27077	35.43141	35.82882	35.55833	35.106
	sd	1.314492	1.357184	1.254027	1.42248	1.542092	1.41047	1.308889	1.308968	1.960973	1.227021

WW	mean	159.9981	158.6019	159.5693	161.8483	153.373	157.9085	155.9706	163.3065	168.145	146.32
	sd	25.73876	23.05114	25.83338	25.4914	22.74741	29.62477	22.90572	25.20128	28.1597	6.414924
WWP	mean	13.15442	13.26556	13.15708	13.21167	13.008	13.03615	13.14237	13.42529	13.19833	13.146
	sd	0.811505	0.80489	0.848744	0.728358	0.646302	0.910966	0.821385	0.929228	0.339848	0.666093
AFW	mean	34.74129	31.14556	35.42891	33.33523	31.858	37.62583	35.00986	29.60118	35.31167	36.662
	sd	12.41153	10.59037	12.38824	12.6817	10.03452	14.57707	11.9022	11.94882	11.06957	10.73175
AFWP	mean	2.819081	2.59	2.877573	2.695981	2.68	3.009167	2.888519	2.415294	2.826667	3.18
	sd	1.036307	0.970985	1.030064	1.072557	0.869265	1.112854	0.991848	1.055534	1.129401	0.910027
